# A basal-like breast cancer-specific role for SRF–IL6 in YAP-induced cancer stemness

**DOI:** 10.1038/ncomms10186

**Published:** 2015-12-16

**Authors:** Tackhoon Kim, Suk-Jin Yang, Daehee Hwang, Jinhoi Song, Minchul Kim, Sang Kyum Kim, Keunsoo Kang, Jaebum Ahn, Daeyoup Lee, Mi-young Kim, Seyun Kim, Ja Seung Koo, Sang Seok Koh, Seon-Young Kim, Dae-Sik Lim

**Affiliations:** 1 Department of Biological Sciences, Korea Advanced Institute of Science and Technology, Daejeon 305-701, Korea; 2 National Creative Research Center for Cell Division and Differentiation, Korea Advanced Institute of Science and Technology, Daejeon 305-701, Korea; 3 Medical Genomics Research Center, Korea Research Institute of Bioscience and Biotechnology, Daejeon 305-806, Korea; 4 Therapeutic Antibody Research Center, Korea Research Institute of Bioscience and Biotechnology, Daejeon 305-806, Korea; 5 Department of Pathology, Yonsei University College of Medicine, Seoul 120-752, Korea; 6 Department of Biological Sciences, Dong-A University, Busan 604-714, Korea

## Abstract

The switch between stem/progenitor cell expansion and differentiation is critical for organ homeostasis. The mammalian Hippo pathway effector and oncoprotein YAP expands undifferentiated stem/progenitor cells in various tissues. However, the YAP-associated transcription factors and downstream targets underlying this stemness-promoting activity are poorly understood. Here we show that the SRF–IL6 axis is the critical mediator of YAP-induced stemness in mammary epithelial cells and breast cancer. Specifically, serum response factor (SRF)-mediated binding and recruitment of YAP to mammary stem cell (MaSC) signature-gene promoters induce numerous MaSC signature genes, among which the target interleukin (IL)-6 is critical for YAP-induced stemness. High SRF–YAP/TAZ expression is correlated with IL6-enriched MaSC/basal-like breast cancer (BLBC). Finally, we show that this high SRF expression enables YAP to more efficiently induce IL6 and stemness in BLBC compared with luminal-type breast cancer. Collectively, our results establish the importance of SRF–YAP–IL6 signalling in promoting MaSC-like properties in a BLBC-specific manner.

## Abstract (web summary)

The downstream effector of the Hippo pathway, YAP, is a critical regulator of both normal and cancer stem cell properties. In this study, the authors show that in basal-like breast tumors YAP forms a complex with SRF and together they induce a mammary stem cell gene signature through the transcriptional activation of *IL-6*.

## Graphical abstract

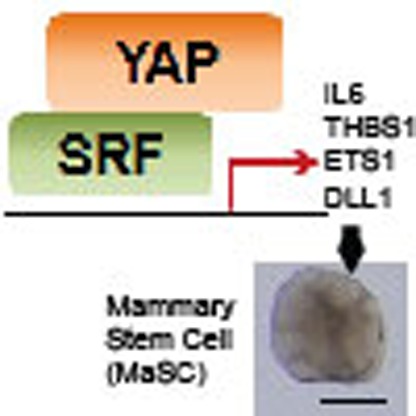

Adult stem cell regulation has been the subject of intense study in recent years. Adult stem cells have been detected in various organs, including the intestine and mammary gland^1^,^2^. These adult stem cells play a critical role in maintaining organ homeostasis, enabling tissue regeneration after organ injury. Adult stem cells also are important in cancer development and progression, with a number of studies demonstrating that tumour-initiating cells share many molecular and cellular characteristics in common with adult and embryonic stem cells^3^. This commonality places studies of adult stem cells at the crossroads of understanding both tissue regeneration and cancer mechanisms. Importantly, targeting tumour-initiating cells is considered a promising anticancer strategy; thus, understanding the regulation of adult stem cells may ultimately bear successful therapy.

The transcriptional co-activator YAP (Yes-associated protein), a downstream effector of the newly emerging Hippo pathway, has recently come to the fore as a critical regulator of tissue regeneration, cancer and adult stem cells. YAP is a putative oncogene located in the 11q22 amplicon found in various types of cancers^4^. Studies on YAP transgenic mice, YAP-knockout mice and Hippo pathway-knockout mice have collectively revealed that YAP is required for adult stem cell activation during tissue damage, and shown that aberrant YAP activation expands epithelial stem/progenitor cells *in vivo*^5^,^6^,^7^,^8^. These findings suggest that understanding the mechanism underlying stemness induction by YAP may shed light on the mechanism responsible for the development of poorly differentiated cancers. It may also suggest effective therapies against cancers overexpressing YAP, which is correlated with poor prognosis^9^. However, mechanisms downstream of YAP remain unclear.

A number of upstream cues that modulate YAP activity have been reported^10^. YAP is mainly inhibited by phosphorylation by LATS kinase, which in turn is regulated by various stimuli, including the Hippo pathway, G-protein-coupled receptor activation and actomyosin tension^11^,^12^,^13^. YAP is the critical nuclear target that translates serum-borne mitogens and matrix-stiffness signals into cell proliferation, invasion, epithelial–mesenchymal transition (EMT) and stemness. Interestingly, the regulation and function of YAP is similar to that of serum response factor (SRF). SRF is activated by serum and is also known to be activated by actin cytoskeleton assembly^14^. SRF is also associated with an EMT-like phenotype in hepatocellular carcinoma (HCC) and skin cancer^15^,^16^, and promotes cancer metastasis^17^ and self-renewal of tumour-initiating breast cancer cells^18^. These observations suggest that YAP and SRF may be functionally related. In line with this, a recent study has shown that SRF and YAP targets largely overlap^19^.

In this study, we find that YAP induces numerous mammary stem cell (MaSC) signature genes. We also determine that SRF is the transcription factor that is critical for YAP-induced transcription of MaSC signature genes, and interleukin (IL)-6 is a critical transcriptional target for the induction of MaSC-like properties. SRF–YAP–IL6 signalling is enriched in MaSC/progenitor cell-like basal-like breast cancer (BLBC) in patients' samples and is required for generation of cancer stem cells (CSCs) and cancer relapse specifically in BLBC. Taken together with the previous discovery of the cytokine Unpaired as an effector of yorkie in flies^20^, these findings identify IL6 as the first universal transcriptional target of YAP involved in promoting stemness that is conserved from flies to humans.

## Results

### YAP induces expression of MaSC signature genes

**Figure 1:**
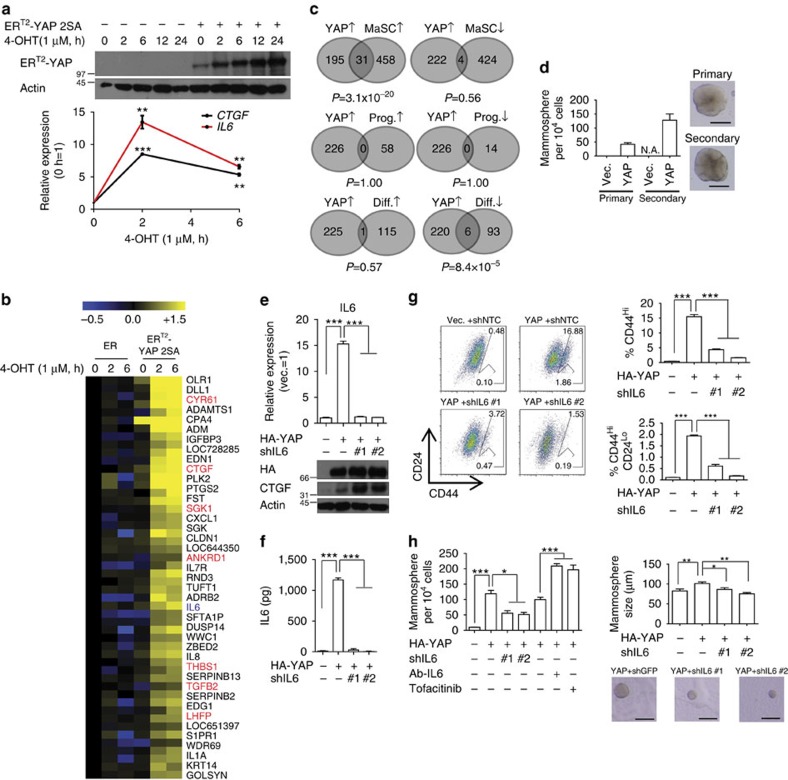
IL6 is required for YAP induction of MaSC-like properties. (**a**) Western blot and quantitative reverse transcriptase–PCR (qRT–PCR) analyses of MCF-10A ER^T2^ and MCF-10A ER^T2^-YAP 2SA cells treated with 4-OHT for the indicated times. (**b**) Heatmap representation of a microarray analysis of MCF-10A ER^T2^ and MCF-10A ER^T2^-YAP 2SA cells treated with 4-OHT. The top 41 genes whose expression in MCF-10A ER^T2^-YAP 2SA cells was significantly changed by the 4-OHT treatment are shown. Refer to Supplementary Data 1 for list of YAP-induced genes. (**c**) Overlap of Venn diagrams of the set of genes enriched by induction of YAP (YAP↑) and gene sets enriched (↑) or depleted (↓) in MaSCs, luminal progenitor cells (Prog.) or differentiated luminal cells (Diff.). *P* values were calculated using Fisher's exact test. (**d**) Number of mammospheres formed by MCF-10A cells expressing indicated gene, and a representative picture of a mammosphere (*n*=3 experiments, scale bar, 200 μm). (**e**–**h**) MCF-10A cells were infected with the indicated retroviruses. shRNA against green fluorescent protein was used as a control, non-targeting shRNA. (**e**) Western blot and qRT–PCR analyses (*n*=2 replicates). (**f**) ELISA for secreted IL6 (*n*=3 replicates). (**g**) Representative FACS plot and statistical analysis of CD44 and CD24 antigen expression (*n*=3 experiments). (**h**) Statistical analyses of the number and sizes of mammospheres formed by YAP-overexpressing cells treated with shRNA against IL6, neutralizing antibody against IL6 (Ab-IL6) or 200 nM tofacitinib (JAK inhibitor). Bottom panel shows representative pictures of mammospheres formed from MCF-10A cells infected with the indicated retroviruses (*n*=3 experiments for number of mammosphere, *n*>50 mammospheres for sizing). Scale bar, 200 μm. Data are presented as means±s.e.m. (*P<0.05, **P<0.01, ***P<0.001, Student's *t*-test used in all analyses unless otherwise indicated).

Microarray analyses using cells/tissues that overexpress YAP have revealed many transcriptional targets of YAP^21^,^22^. However, because YAP induces transformation of noncancerous cells, it is likely that many of the previously identified YAP targets are consequences of the transforming property of YAP. To better define downstream signalling events of YAP activation, we first utilized immortalized mammary epithelial MCF-10A cells expressing a tamoxifen-inducible, hyperactive (S127/381A; two major LATS phosphorylation sites are mutated alanine) YAP mutant (MCF-10A ER^T2^-YAP 2SA). Maximally active YAP 5SA mutant (all five LATS phosphorylation sites are mutated to alanine) was not used because it caused leaky transforming activity without treatment of 4-hydroxytamoxifen. Treatment of MCF-10A ER^T2^-YAP 2SA cells with 4-OHT activated YAP and upregulated the YAP transcriptional target CTGF (connective tissue growth factor) within 2 h (Fig. 1a). Notably, whereas ER^T2^-YAP 2SA exhibited some leaky expression, it was functionally inactive, probably owing to cytoplasmic sequestration by heat-shock protein binding^23^. Our findings support this, demonstrating that vehicle-treated MCF-10A ER^T2^-YAP 2SA cells were unable to upregulate the YAP target gene *CTGF*, induce serum- or anchorage-independent growth or promote expression of the YAP signature transcriptome (Supplementary Fig. 1a–d). Microarray analyses of RNAs from MCF-10A ER^T2^-YAP 2SA and MCF-10A ER^T2^ cells at 2 and 6 h after treatment with 4-OHT identified 226 out of 31,333 (0.7%) nonredundant genes in the microarray as YAP-induced target genes (Fig. 1b and Supplementary Data 1, see Methods for definition of YAP target genes). Although this list of genes may contain indirect consequences of YAP activation, it represents a set of more stringent, early-responsive YAP targets. Notably, of the previously documented YAP signature genes, 24% (16/68) were confirmed in our list of transcriptional targets (Fig. 1b and Supplementary Fig. 1g). A gene ontology analysis of genes exclusively enriched in 4-OHT-treated MCF-10A ER^T2^-YAP 2SA cells classified YAP targets as genes involved in diverse cancers (Supplementary Fig. 1h). Notably, many of the YAP targets included components of the Hippo pathway and secreted oncogenic molecules, such as IL6, IL8, CXCL1 (chemokine [C-X-C motif] ligand 1), CTGF and CYR61 (cysteine-rich angiogenic inducer 61), suggesting a possible negative feedback mechanism^24^ and non-cell-autonomous role of YAP in tumorigenesis, respectively^25^,^26^ (Supplementary Fig. 1e,f).

We next questioned whether the transcriptome of YAP-activated cells resembled that of undifferentiated cells. Mammary epithelial cells can generally be classified into three increasingly differentiated types: MaSCs, luminal progenitor cells and mature luminal cells^27^. The YAP target gene set revealed by our microarray analysis was searched for overlap between gene sets enriched or depleted specifically in each cell type established by Visvader and colleagues^28^. Strikingly, the set of identified YAP targets significantly overlapped (6%, 31/489 genes, *P*=3.1 × 10^−20^, Fisher's exact test), with the set of genes enriched in MaSCs and depleted in mature luminal cells (Fig. 1c). In contrast to the exclusive function of TAZ in luminal-basal cell plasticity^29^, both YAP and TAZ activated transcription of MaSC signature genes, suggesting that these factors are functionally redundant in promoting MaSC-like properties (Supplementary Fig. 2a). Consistent with this, YAP overexpression increased the number of mammospheres (Fig. 1d). Non-hyperactive, wild-type YAP was used in these experiments because hyperactive YAP might promote anchorage-independent growth and confound analysis of mammosphere assays.

We reasoned that IL6 could be responsible for YAP-induced MaSC-like properties, partly because IL6 is enriched in MaSCs and is essential for the maintenance of breast CSCs, but also because the *Drosophila* homologue of YAP–IL6 (yorkie-unpaired) is important for intestinal stem cell activation^20^,^28^,^30^. We confirmed that YAP induced IL6 at the mRNA level (Fig. 1e and Supplementary Fig. 2b) and increased IL6 secretion (Fig. 1f). Depletion of IL6 decreased the proportion of CD44^Hi^/CD24^Lo^ cells and decreased both the number and size of mammospheres (Fig. 1g,h), while increasing CTGF at the post-transcriptional level (Fig. 1e and Supplementary Fig. 3a). Notably, IL6 depletion did not reverse EMT or alter cell proliferation or apoptosis (Supplementary Fig. 3a–c), thus IL6, while not influencing other transforming properties, is specifically involved in promoting MaSC-like property. Depleting the YAP target CTGF failed to attenuate MaSC-like properties (Supplementary Fig. 4). Surprisingly, an IL6-neutralizing antibody and an inhibitor of IL6 downstream JAK signalling increased mammosphere frequency (Fig. 1h). Since IL6 intracellular signalling has been demonstrated in the senescence-associated secretory phenotype^31^, we hypothesized that intracellular IL6 may similarly be responsible for YAP-induced MaSC-like properties. Indeed, although MCF-10A cells expressing nonsecretable mutant IL6 lacking a signal peptide (IL6 ΔS) failed to activate JAK signalling, it generated mammospheres at a frequency comparable to that of MCF-10A cells expressing wild-type IL6 (Supplementary Fig. 3d–f), suggesting the predominant role of intracellular IL6 in promoting MaSC-like property. Accordingly, treatment of recombinant human IL6 failed to increase mammosphere in untransformed MCF-10A cells (Supplementary Fig. 3g). This is in sharp contrast with IL6-JAK signalling being the major determinant of cancer stemness in transformed cells^30^ (Fig. 6g). We are uncertain why inhibition of extracellular IL6 promotes mammosphere formation; the balance between intra- and extracellular IL6 signalling may determine MaSC-like property, where extracellular IL6 signalling may possibly inhibit MaSC-like property.

### TEAD is dispensable for YAP-induced MaSC-like properties

**Figure 2:**
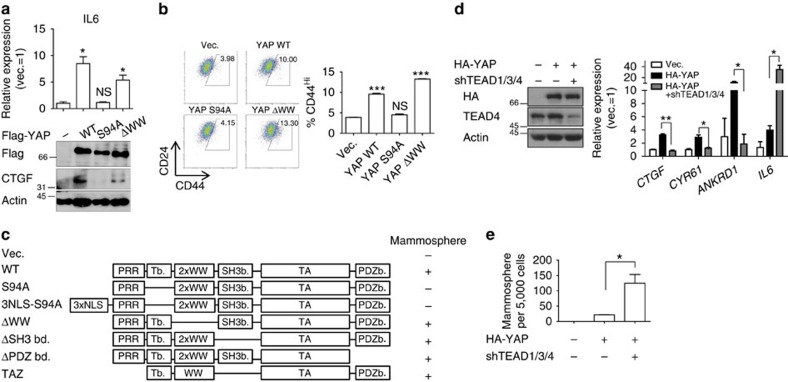
TEAD transcription factor is dispensable for YAP induced MaSC formation. (**a**–**c**) The TEAD-binding domain of YAP is required for induction of MaSC-like properties. (**a**) Western blot and qRT–PCR analyses of cells infected with the indicated YAP mutants (*n*=2 replicates). (**b**) Representative FACS plot and statistical analysis of CD44 and CD24 antigen expression. (**c**) Summary of results from mammosphere assays using MCF-10A cells infected with the indicated YAP mutants. (**d**,**e**) MCF-10A cells expressing YAP were infected with lentivirus expressing TEAD1/3/4 shRNA. (**d**) Western blot and qRT–PCR analyses (*n*=2 replicates). (**e**) Statistical analyses of the number of mammospheres formed by MCF-10A cells expressing the indicated viruses (*n*=3 experiments). Data are presented as means±s.e.m. (*P<0.05, **P<0.01, ***P<0.001; NS, not significant (*P*>0.05), Student's *t*-test used in all analyses).

Next, we asked which transcription factors are responsible for YAP-induced MaSC properties. Since the vast majority of YAP's physical associations with transcription factors depend on TEAD-binding and WW domains^32^, we utilized mutant YAPs that individually lacked each of these protein-interaction domains and examined induction of MaSC properties and expression of IL6 and CTGF as representative MaSC signature genes. Strikingly, whereas YAP ΔWW was fully competent in promoting IL6 and CTGF expression and generating CD44^Hi^ cells, YAP S94A, the TEAD-binding-deficient YAP, was completely unable to do so (Fig. 2a,b). On overexpression of mutant YAPs lacking each of the known protein-interaction motifs in MCF-10A cells, only cells expressing YAP S94A failed to form mammospheres (Fig. 2c). Since TEAD binding may enhance YAP nuclear retention^33^, we were concerned that S94A mutants might not enter the nucleus at all. However, even when YAP S94A was forced into the nucleus by fusion of nuclear localization signal (Supplementary Fig. 5), it failed to generate mammospheres (Fig. 2c).

We next assessed the role of TEAD transcription factors using short hairpin RNA (shRNA)-mediated depletion of TEAD1, -3 and -4, the major forms of TEAD in MCF-10A cells (Supplementary Fig. 6a). Surprisingly, whereas TEAD knockdown effectively downregulated the YAP transcriptional targets CTGF, CYR61 and ANKRD1, it robustly upregulated IL6 expression (Fig. 2d and Supplementary Fig. 6b) and promoted mammosphere formation (Fig. 2e and Supplementary Fig. 6c). To further investigate these results, we performed rescue experiments in which we re-introduced wild-type TEAD2 or one of two inactive TEAD2 mutants—R95K (RK) and a C-terminal deletion mutant (ΔC)—which fail to bind DNA and YAP, respectively^34^,^35^, as naturally occurring shRNA-resistant TEADs. Strikingly, all forms of TEAD2 efficiently downregulated IL6, indicating that TEAD suppresses IL6 by a mechanism independent of functional TEAD–YAP activity. However, whereas wild-type TEAD2 efficiently rescued the mammosphere frequency of TEAD1/3/4-knockdown cells, the TEAD2 RK and ΔC mutants only partially decreased sphere-forming frequency (Supplementary Fig. 6b,c). We speculated that TEAD may interfere with YAP-induced MaSC-like properties through two distinct mechanisms: one involving regulation of IL6 levels by a mechanism independent of TEAD–YAP-mediated transcription and another involving a functional TEAD–YAP transcriptional complex possibly inducing transcription of target genes distinct from SRF–YAP targets (that is, IL6) to suppress MaSC-like property. To confirm this, we overexpressed IL6 that far exceeds IL6 induced by YAP overexpression to prevent TEAD depletion from significantly influencing IL6 levels (Supplementary Fig. 6d). In this setting, TEAD depletion still increases in mammosphere frequency that could only be rescued by expression of wild-type TEAD, suggesting an IL6-independent, TEAD transcription factor activity-dependent mode in the suppression of MaSC-like properties (Supplementary Fig. 6e). These data suggest that an alternative transcription factor that requires the N-terminal TEAD-binding domain of YAP, but is distinct from TEAD, may be responsible for YAP-induced promotion of MaSC properties.

### The SRF–YAP complex is required for MaSC-like properties

**Figure 3:**
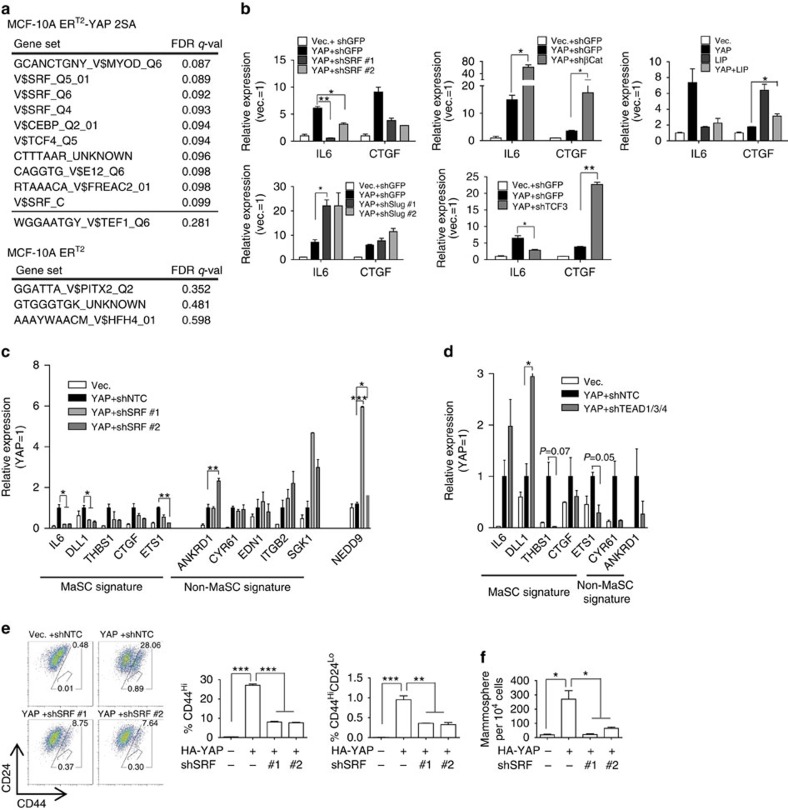
SRF is critical for YAP induction of MaSC-like properties. (**a**) Summary of GSEA analysis for sets of transcription factor targets. The top 10 gene sets enriched in MCF-10A ER^T2^-YAP 2SA cells and top three gene sets enriched in MCF-10A ER^T2^ cells with lowest false discovery rate are shown. Full GSEA analysis results are presented in Supplementary Data 2. (**b**) qRT–PCR analyses of YAP-overexpressing MCF-10A cells infected with shRNA targeting transcription factors or coexpressing the dominant-negative transcription factor LIP (*n*=2 replicates). Refer to Supplementary Fig. 7b for successful expression of shRNA or dominant-negative transcription factor. (**c**,**d**) Distinct transcriptional regulation of YAP target genes by SRF and TEAD. qRT–PCR analyses of MCF-10A cells overexpressing YAP and shRNA against (**c**) SRF (*n*=2 replicates) or (**d**) TEAD1/3/4 (*n*=2 replicates). (**e**,**f**) SRF is required for YAP induction of MaSC-like properties. (**e**) Representative FACS plot and statistical analyses of CD44 and CD24 antigen expression (*n*=3 experiments) and (**f**) the number of mammospheres formed by MCF-10A cells expressing YAP and shRNA against SRF (*n*=3 experiments). Data are presented as means±s.e.m. (*P<0.05, **P<0.01, ***P<0.001, Student's *t*-test used in all analyses).

As an unbiased approach for identifying candidate alternative transcription factor(s) responsible for YAP-induced MaSC properties, we applied a Gene Set Enrichment Analysis (GSEA) to our microarray data for MaSC signature genes^36^. A number of gene sets, including E12/E47 (TCF3), TCF4/LEF1-β-catenin and SRF targets, were significantly enriched in MCF-10A ER^T2^-YAP 2SA cells (false discovery rate *q*-value<0.25; Fig. 3a and Supplementary Fig. 7a and Supplementary Data 2). Notably, TEAD target enrichment failed to reach statistical significance (false discovery rate=0.28). We first examined whether inactivation of these transcription factors influenced the expression of MaSC genes by quantifying IL6 and CTGF mRNA as representative MaSC genes. Strikingly, SRF was the only factor whose depletion significantly decreased expression of both genes (Fig. 3b and Supplementary Fig. 7b). Depletion of SRF downregulated many YAP-induced MaSC signature genes, including *IL6*, *CTGF*, *THBS1* (thrombospondin 1), *ETS1* and *DLL1* (delta-like 1; Fig. 3c). Expression of NEDD9, a target of SRF–MRTF previously reported to promote breast cancer-initiating cells^18^, was not affected by YAP overexpression (Fig. 3c). Interestingly, SRF knockdown did not affect expression of other YAP target genes that are not MaSC signature genes, such as CYR61 and ANKRD1. This was in sharp contrast with TEAD knockdown, which markedly decreased many YAP target genes regardless of their MaSC signature-gene status (Fig. 3d). Intriguingly, IL6 and DLL1 were not downregulated by TEAD knockdown. This suggests that SRF specifically regulates expression of MaSC signature genes, and further that YAP target genes that are exclusively regulated by SRF and not by TEAD may represent a gene subset critical for YAP-induced MaSC-like properties. Importantly, SRF knockdown attenuated the YAP-induced increase in the CD44^Hi^/CD24^Lo^ cell population and mammosphere formation (Fig. 3e,f). SRF overexpression in MCF-10A cells caused cell death, perhaps by a defence mechanism to control oncogenic dose. However, in case of fully transformed, metastatic 4T1 cells, we found that YAP was also required for SRF-induced MaSC-like properties (Supplementary Fig. 7c); moreover, SRF and YAP synergistically induced MaSC-like properties in 4T1 cells (Supplementary Fig. 7d). Finally, SRF depletion eliminated mammosphere formation in both MCF-10A and 4T1 cells (Supplementary Fig. 7e). These data suggest that SRF is the critical transcription factor responsible for YAP induction of MaSC signature-gene expression and MaSC-like properties.

**Figure 4:**
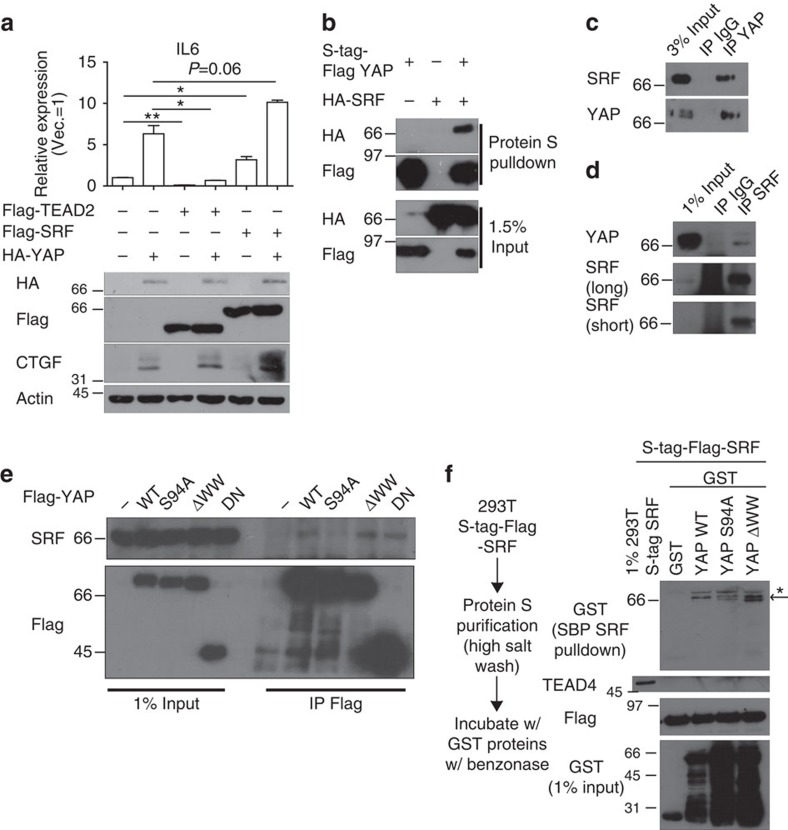
SRF interacts with YAP. (**a**) Western blot and qRT–PCR analyses (*n*=2 replicates) of MCF-10A cells coexpressing YAP and TEAD2/SRF. Note that SRF, but not TEAD2, synergized with YAP in promoting IL6 expression. (**b**) Western blot showing YAP–SRF co-immunoprecipitation with SRF- and/or YAP-overexpressing 293T cell extract crosslinked with dithiobis(succinimidyl propionate) (DSP). (**c**,**d**) Western blot showing endogenous YAP–SRF co-immunoprecipitation with (**c**) YAP antibody or (**d**) SRF antibody in 293T cell extract crosslinked with DSP. (**e**) Western blot showing co-immunoprecipitation with the indicated Flag-tagged mutant YAP and SRF. Note that YAP S94A fails to bind SRF. (**f**) *In vitro* pulldown assay with purified S-tag-Flag-SRF and bacterially purified GST-tagged YAP mutants. Asterisk indicates nonspecific bands, and the arrow indicates the desired band.

**Figure 5:**
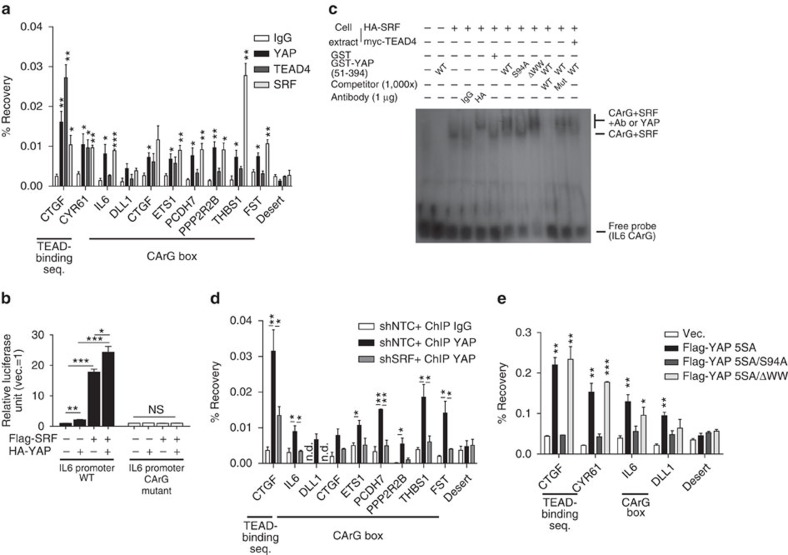
Interaction with SRF recruits YAP to MaSC signature-gene promoters. (**a**) ChIP–qPCR analyses of the indicated transcriptional regulators (*n*=3 experiments). (**b**) Luciferase assay of the *IL6* promoter CArG box (*n*=3 experiments). (**c**) EMSA assay of the *IL6* promoter CArG box using 293T extracts expressing indicated genes and GST-tagged proteins. (**d**) ChIP–qPCR analyses of the indicated cells (*n*=3 experiments). (**e**) ChIP–qPCR analyses of the TEAD-binding boxes of *CTGF/CYR61* promoters and CArG boxes of *IL6/DLL1* promoters in MCF-10A cells expressing the indicated mutant YAP (*n*=3 experiments). YAP 5SA mutants are used to maximize chromatin binding. Data are presented as means±s.e.m. (*P<0.05, **P<0.01, ***P<0.001, NS, not significant (*P*>0.05), Student's *t*-test used in all analyses).

We reasoned that SRF, but not TEAD, forms a transcriptional complex with YAP at MaSC signature-gene promoters. Consistent with this interpretation, we found that coexpression of SRF, but not TEAD, with YAP synergistically upregulated IL6 and CTGF (Fig. 4a). Although synergistic CTGF induction by SRF and YAP was reversed by TEAD depletion, SRF–YAP could independently increase CTGF because it is already a well-established target gene of SRF (Supplementary Fig. 7f). Co-immunoprecipitation assays revealed that SRF associates with YAP at exogenous and endogenous levels independent of contaminating DNA (Fig. 4b–d and Supplementary Fig. 7g). Domain mapping of YAP–SRF interactions revealed that interactions with SRF are attenuated in the YAP S94A mutant (Fig. 4e,f). This may explain why the YAP S94A mutant failed to induce MaSC-like properties, whereas TEAD transcription factors were dispensable for this. Next, we examined the recruitment of YAP to SRF-binding DNA motif, the CArG boxes, in promoters of MaSC signature genes, including *IL6*, *CTGF, PCDH7*, *THBS1*, *PPP2R2B*, *ETS1* and *DLL1* (Supplementary Fig. 8a), using chromatin immunoprecipitation (ChIP) assays. Intriguingly, YAP was significantly enriched at CArG boxes within MaSC gene promoters where SRF was also enriched (Fig. 5a). We further confirmed that SRF and YAP synergized in activating the CArG box-containing *IL6* promoter using luciferase assays (Fig. 5b) and detected a DNA–SRF–YAP complex that was attenuated by YAP S94A mutation using electromobility shift assay (EMSA; Fig. 5c). Consistently, SRF depletion also mitigated YAP enrichment at promoters of the above-mentioned MaSC signature genes (Fig. 5d). YAP was also enriched at CArG boxes in YAP target genes whose expression was unaffected by SRF depletion, such as *CYR61* and *ANKRD1*, and this enrichment was diminished by SRF depletion (Supplementary Fig. 8c,f). We believe that, although these two genes can indeed be regulated by SRF–YAP, the regulation by TEAD–YAP may be predominant. Finally, in line with results of our YAP–SRF interaction domain mapping, the YAP S94A mutant failed to localize not only to TEAD-binding boxes in *CTGF* and *CYR61* promoters, but also to CArG boxes in *IL6* and *DLL1* promoters (Fig. 5e). It should be noted that SRF and YAP interacted *in vitro* without detectable TEAD4 protein (Fig. 4f), and exogenous TEAD4 failed to further shift the DNA–SRF–YAP complex in EMSA assay (Fig. 5c, compare lanes 7 and 12). These lines of evidence indicate that the SRF–YAP complex may exist independent of TEAD. Consistently, TEAD depletion had little effect on IL6 promoter activity and YAP enrichment at MaSC signature-gene promoters (Supplementary Fig. 8d,e). Collectively, these data suggest that SRF is the critical transcription factor responsible for YAP induction of MaSC signature-gene expression and MaSC formation.

MRTF (myocardin-related transcription factor) family proteins are well known as SRF co-activators. Thus, we examined the possibility that MRTF was activated by cytoskeletal changes induced by YAP. Notably, YAP overexpression did not change MRTF protein localization, previously known SRF–MRTF target genes, or F-actin/G-actin ratio, which regulates MRTF activity (Supplementary Fig. 9a–c). YAP enrichment was not detected at known SRF–MRTF-binding sites (Supplementary Fig. 8b), and TEAD depletion did not change SRF–MRTF-responsive reporter- or *IL6* promoter–reporter activities (Supplementary Figs 8d and 9d). Finally, MRTF depletion had little effect on YAP-induced MaSC signature-gene expression or mammosphere frequency (Supplementary Fig. 9e–g). This suggests that SRF–YAP-induced MaSC signature-gene expression is independent of SRF–MRTF activity.

### SRF–YAP/TAZ–IL6 promotes Ras-induced CSC formation

**Figure 6:**
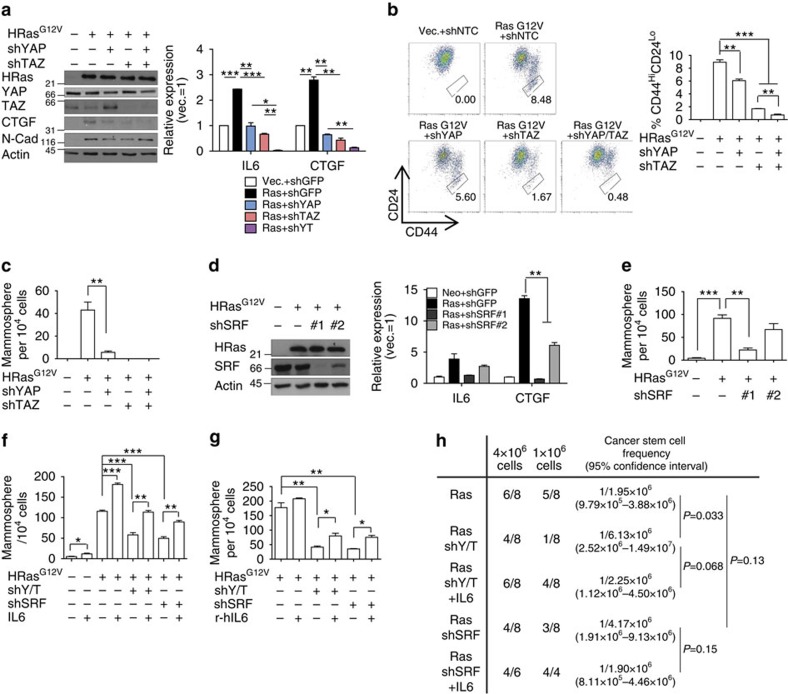
SRF–YAP–IL6 signalling is required for CSC formation. (**a**–**c**) YAP/TAZ knockdown decreases IL6 expression and CSC formation. MCF-10A cells were transformed with a constitutively active form of H-Ras (HRas^G12V^) and then transduced with shRNA against the indicated genes. (**a**) Western blot and qRT–PCR analyses (*n*=2 replicates), (**b**) representative FACS plot and statistical analyses of CD44 and CD24 antigen expression (*n*=3 experiments) and (**c**) statistical analyses of the number of mammospheres formed by MCF-10A cells expressing HRasG12V together with shRNA against YAP and/or TAZ (*n*=3 experiments). (**d**,**e**) MCF-10A H-Ras^G12V^ cells were infected with shRNA against SRF. (**d**) Western blot and qRT–PCR analyses (*n*=2 replicates) and (**e**) statistical analyses of the number of mammospheres formed by cells infected with the indicated viruses (*n*=3 experiments). (**f**,**g**) MCF-10A H-Ras^G12V^ cells infected with shRNA against YAP/TAZ or SRF were (**f**) infected with IL6-expressing retrovirus or (**g**) were treated with recombinant IL6 (r-hIL6; 5 ng ml^−1^). Statistical analyses of the number of mammospheres formed by cells infected with the indicated viruses (*n*=3 experiments). (**h**) Limiting dilution xenograft assays of cells generated in **f**. Data are presented as means±s.e.m. (*P<0.05, **P<0.01, ***P<0.001, Student's *t*-test used in all analyses).

Our demonstration that SRF–YAP–IL6 signalling is important for induction of MaSC-like properties suggests that this pathway is required for CSC formation. To test this, we used H-Ras^G12V^-transformed MCF-10A cells, which have high IL6 levels for CSC maintenance (Supplementary Fig. 10a–c). Notably, CTGF was dispensable for H-Ras^G12V^-induced CSC formation (Supplementary Fig. 10d,e). Strikingly, YAP/TAZ knockdown reduced IL6 expression and decreased the CD44^Hi^/CD24^Lo^ cell population and tumoursphere-formation frequency in MCF-10A H-Ras^G12V^ cells (Fig. 6a–c). We observed similar decreases in IL6 expression and subsequent tumoursphere frequency by YAP/TAZ depletion in two other BLBC cell lines: MDA-MB-231 and Hs578T (Supplementary Fig. 10f). SRF knockdown similarly significantly decreased the IL6 expression level and tumoursphere-formation frequency in MCF-10A H-Ras^G12V^ cells (Fig. 6d,e). Notably, TEAD knockdown had little effect on tumoursphere-formation frequency in MCF-10A H-Ras^G12V^ cells, indicating that TEADs are dispensable for CSC formation (Supplementary Fig. 10g). Furthermore, restoration of IL6 expression or treatment with recombinant IL6 in YAP/TAZ- or SRF-depleted MCF-10A H-Ras^G12V^ was sufficient to restore the frequency of tumoursphere formation, confirming that IL6 is the critical factor for SRF–YAP/TAZ-induced CSC formation (Fig. 6f,g). Using a limiting dilution xenograft assay, we also found that IL6 overexpression partially rescued the decrease in tumour-initiating activity of MCF-10A H-Ras^G12V^ cells induced by YAP/TAZ or SRF knockdown (Fig. 6h).

**Figure 7:**
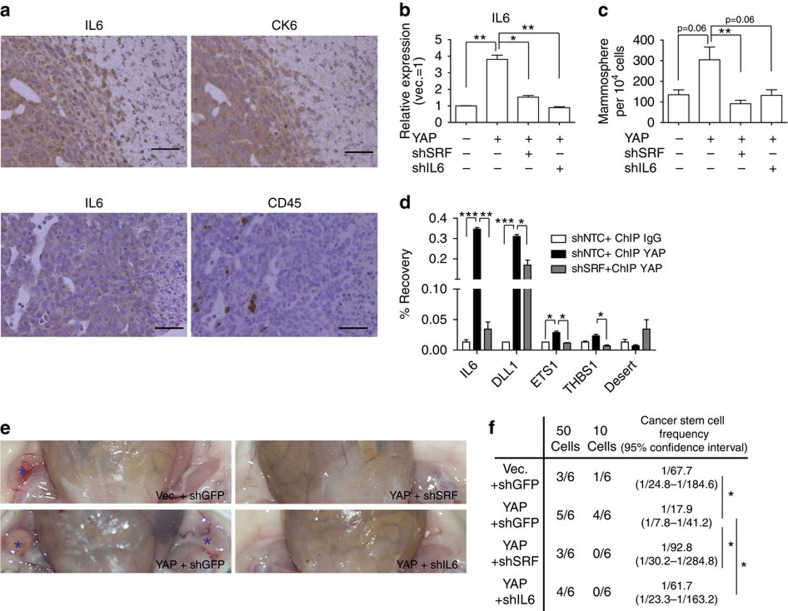
SRF–YAP–IL6 signalling promotes cancer stemness *in vivo*. (**a**) Immunohistochemical analysis of serial sections of 4T1 syngeneic graft tumours. Scale bar, 100 μm. (**b**,**c**) 4T1 cells were infected with retrovirus overexpressing YAP and lentivirus expressing shRNA against the indicated genes. (**b**) qRT–PCR analysis of IL6 expression (*n*=2 replicates) and (**c**) quantification of the number of mammospheres (*n*=3 experiments). (**d**) ChIP–qPCR analyses of 4T1 cells infected with the indicated viruses (*n*=3 experiments). (**e**,**f**) Cells generated in **b** were injected into BALB/c mice. (**e**) Representative tumour image. Blue asterisks indicate tumours. (**f**) Summary data of tumour-initiation frequency. Data are presented as means±s.e.m. (*P<0.05, **P<0.01, ***P<0.001, Student's *t*-test used in all analyses).

Since IL6 cytokine is also expressed in immune cells, xenografts from immune-deficient mice may not faithfully reflect the *in vivo* significance of SRF–YAP–IL6 signalling in tumour initiation. To address this, we used a 4T1 syngeneic tumour graft model in immune-competent BALB/c mice. Immunohistochemical analyses of serial tumour sections revealed that, whereas IL6-expressing regions largely overlapped with those for cytokeratin 6, a 4T1 cancer cell marker, they did not significantly overlap with those for CD45, an immune cell marker (Fig. 7a). Taken together with our evidence that IL6 intracellular signalling may induce MaSC-like properties, these findings suggest that tumour cell-derived IL6 is a major mediator of CSC properties. We further examined the significance of the SRF–YAP–IL6 signalling axis *in vivo* using the 4T1 breast cancer cell line syngeneic graft model. YAP increased IL6 in an SRF-dependent manner in 4T1 cells, and depletion of either SRF or IL6 decreased tumoursphere frequency (Fig. 7b,c). YAP was also recruited to CArG boxes in MaSC gene promoters in an SRF-dependent manner (Fig. 7d). Syngeneic graft assays again showed that YAP overexpression increased tumour-initiating cell frequency, and SRF or IL6 depletion decreased it (Fig. 7e,f). These results confirm that IL6 is a critical factor in SRF- and YAP/TAZ-dependent induction of CSC formation *in vivo*.

### SRF–YAP/TAZ–IL6 is enriched in undifferentiated BLBC

**Figure 8:**
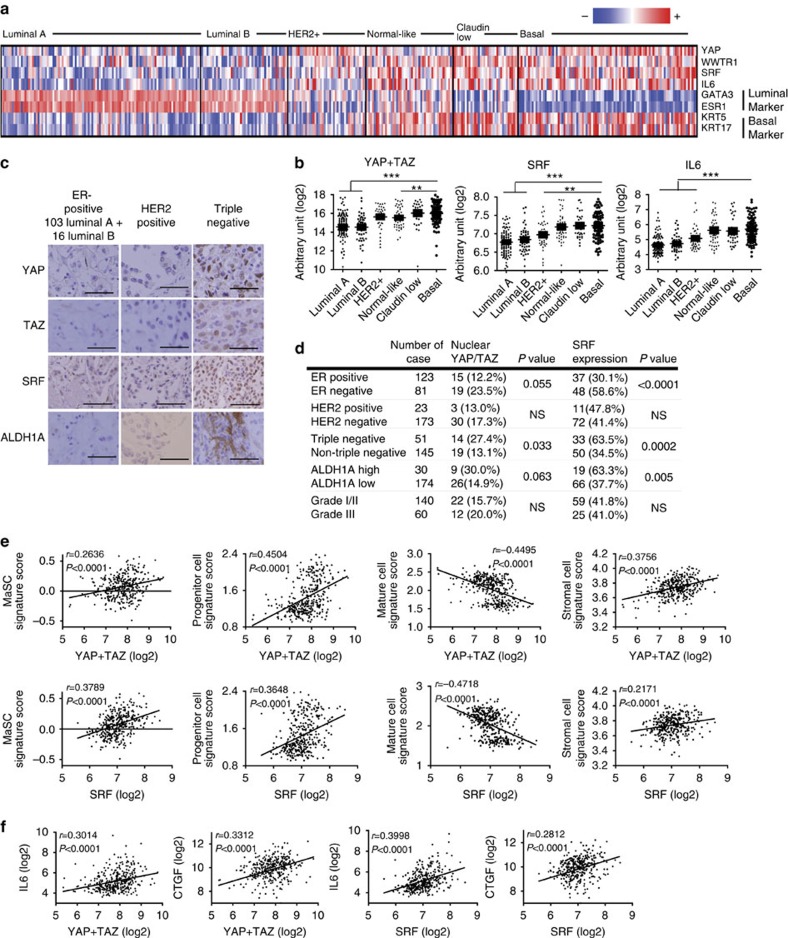
SRF–YAP–IL6 signalling is upregulated in basal-type breast cancer. (**a**,**b**) SRF–YAP/TAZ–IL6 are highly expressed BLBC. (**a**) Heatmap analysis (**b**) and statistical analysis of the expression of the indicated genes in a panel of breast cancers (GSE31448; *n*>27 for each subtype). (**c**,**d**) A tissue microarray of breast cancers was stained with antibodies against the indicated proteins. (**c**) Representative immunohistochemical data for different types of breast cancers. Scale bar, 100 μm. (**d**) Summary and statistical analysis of tissue microarray data. *P* values were calculated using Fisher's exact test. (**e**) Scatter plot of signature score for the indicated cell types against YAP+TAZ or SRF expression levels (*n*=357; correlation tested using Pearson's correlation coefficient, *r*). (**f**) Scatter plot of IL6 and CTGF expression against YAP+TAZ or SRF expression levels (*n*=357; correlation tested using Pearson's correlation coefficient, *r*). Data are presented as means±s.e.m. (**P<0.01, ***P<0.001, NS, not significant (*P*>0.05), Student's *t*-test used in all analyses).

To extend our findings to human breast cancer, we examined expression levels of SRF, YAP/TAZ and IL6 in human breast cancer patients by analysing microarray data from various breast cancer study cohorts. SRF, YAP, TAZ (WWTR1) and IL6 are consistently upregulated in BLBC, normal-like breast cancer and claudin-low subtypes (Fig. 8a,b). Although the claudin-low subtype is known to exhibit the highest similarity to MaSC and thus is expected to have the highest activity of YAP/TAZ, this subtype did not have higher YAP/TAZ mRNA levels compared with BLBC, which is known to be derived from luminal progenitor cells^37^. The claudin-low subtype might have higher post-transcriptional YAP/TAZ activity owing to nuclear localization downstream of EMT^9^. We again confirmed the importance of SRF and YAP/TAZ in breast cancer stemness using tissue arrays. SRF expression and nuclear YAP/TAZ were clearly associated with triple-negative BLBC and ALDH1A1 (aldehyde dehydrogenase 1A1)-positive breast CSCs^38^ (Fig. 8c,d).

Next, for each patient studied in microarrays, we determined signature scores for four cell types—MaSCs, luminal progenitor cells, mature luminal cells and stromal cells—as defined by Visvader and colleagues^37^, and examined their correlation with SRF and YAP/TAZ expression levels. Strikingly, both SRF and YAP/TAZ expression levels were positively correlated with MaSC, luminal progenitor cell and stromal cell signatures, and negatively correlated with the mature luminal cell signature (Fig. 8e). Finally, we found a significant positive correlation between YAP/TAZ and SRF expression and the levels of IL6, implying that IL6 is induced by SRF and YAP/TAZ *in vivo* (Fig. 8f). All of the foregoing observations were similarly demonstrated in other independent breast cancer cohorts (Supplementary Fig. 13a–d). These data suggest that upregulation of SRF–YAP–IL6 signalling is associated with poorly differentiated BLBC with MaSC-like properties, further highlighting the importance of these genes in breast cancer.

Notably, MRTF family genes exhibited a contrasting pattern of expression. MRTFA was enriched in BLBC and was positively correlated with MaSC/progenitor cell signature and IL6 expression, whereas MRTFB was enriched in luminal-type breast cancer and was negatively correlated with MaSC/progenitor cell signature and IL6 expression (Supplementary Fig. 11). Although MRTFA, rather than MRTFB, may specifically be involved in MaSC-like property, our finding that MRTFA depletion did not attenuate MaSC-like property (Supplementary Fig. 9e–g) suggests that MRTFA enrichment in BLBC may not reflect the causal relationship with MaSC-like property. Future studies should reveal the cause of distinct correlation of MRTF gene expressions.

### YAP selectively induces cancer stemness in BLBC

**Figure 9:**
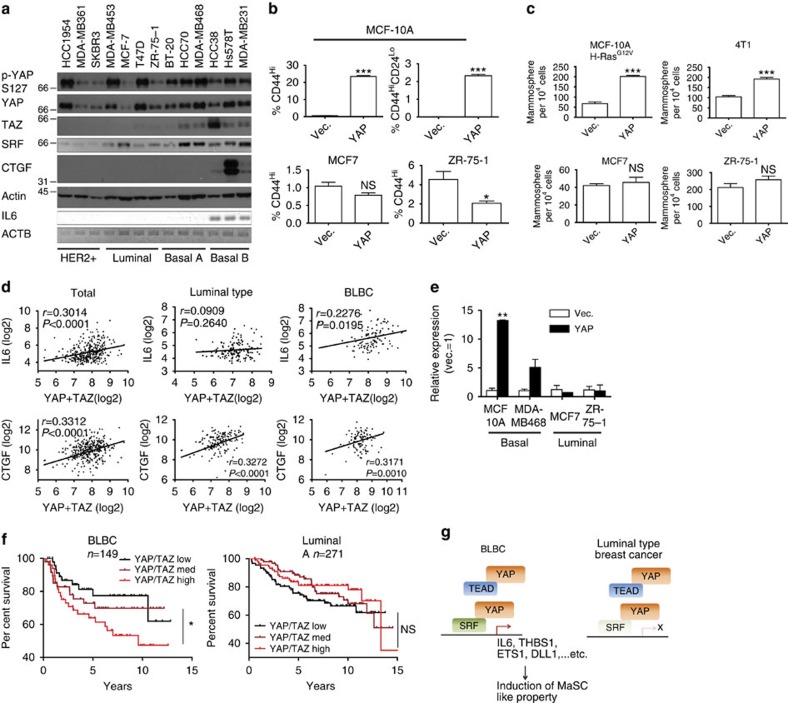
YAP is a BLBC-specific inducer of stemness. (**a**) A panel of breast cancer cell lines was analysed using semiquantitative RT–PCR and western blot analysis using the indicated antibodies. (**b**) Statistical analyses of CD44 and CD24 antigen expression in basal and luminal cell lines overexpressing YAP, determined by FACS (*n*=3 experiments). (**c**) Mammosphere assay of cells generated in **b** (*n*=3 experiments). (**d**) Scatter plot of IL6 and CTGF expression versus YAP+TAZ expression level in luminal cancer and BLBC. Note that IL6 and YAP/TAZ expression levels are not significantly correlated in luminal cancer (*n*>100 for each subtype; correlation tested using Pearson's correlation coefficient, *r*). (**e**) RT-qPCR analyses of IL6 mRNA expression for the indicated luminal and basal cancer cell lines overexpressing YAP (*n*=3 replicates). (**f**) Kaplan–Meier curves for luminal-type breast cancer patients and BLBC patients with different levels of YAP/TAZ expression (log-rank test). Refer to Supplementary Fig. 12f for survival curves for other breast cancer subtypes. (**g**) Molecular model of BLBC-specific YAP induction of MaSC properties. Data are presented as means±s.e.m. (*P<0.05, **P<0.01, ***P<0.001; NS, not significant (*P*>0.05), Student's *t*-test used in all analyses).

Intrigued by our finding that SRF, which is essential for YAP-induced MaSC-like properties, is highly expressed in BLBC but not in luminal-type breast cancer, we investigated the possibility that YAP might activate IL6 expression and MaSC-like properties specifically in BLBC. Indeed, an expression analysis of various breast cancer cell lines revealed that SRF, YAP/TAZ and IL6 are all upregulated in BLBC cell lines compared with other types of breast cancer (Fig. 9a). Basal B-type cancer cell lines with a mesenchymal phenotype showed the highest expression of the YAP targets, IL6 and CTGF. This reflects the hyperactivity and nuclear localization of YAP (Supplementary Fig. 12) in these cell lines, possibly because of EMT^9^. Consistent with our hypothesis, YAP overexpression in basal cells or BLBCs, such as MCF-10A, MCF-10A H-Ras^G12V^ and 4T1, increased IL6 expression, the percentage of cells with a CD44^Hi^CD24^Lo^ surface antigen profile and mammosphere frequency, whereas it failed to increase IL6 and MaSC-like properties in luminal-type cancer cells, such as MCF-7 and ZR-75-1 (Fig. 9b,c,e). Coexpression of SRF and YAP/TAZ in many of the luminal-type cancer cell lines was, nevertheless, insufficient to induce MaSC-like properties, suggesting that SRF–YAP is necessary, but not sufficient, for manifestation of MaSC-like properties (Supplementary Fig. 10h,i). Consistent with our cell line study, IL6 expression was significantly correlated with YAP/TAZ expression in BLBC, but not in luminal-type breast cancer, indicating that IL6 could be a BLBC-specific transcriptional target of YAP (Fig. 9d and Supplementary Fig. 13e). This pattern is in sharp contrast with that of CTGF, a well-known universal target of YAP, whose expression was positively correlated with YAP/TAZ expression regardless of breast cancer subtype.

CSCs are thought to be the cell of origin for cancer relapse after chemotherapy. Therefore, we hypothesized that specific induction of stemness in BLBC by YAP promotes cancer relapse. To this end, we examined the correlation between YAP/TAZ expression levels and patient outcome in different breast cancer subtypes. Strikingly, we found that, whereas YAP/TAZ expression had little correlation with patient prognosis in other subtypes of breast cancer, it was significantly correlated with shorter relapse-free survival time for BLBC patients (Fig. 9f and Supplementary Fig. 13f). Collectively, these findings indicate that the high expression of SRF in BLBC provides a signalling environment in which YAP can more efficiently induce MaSC signature genes and MaSC-like properties. Therefore, YAP could be a BLBC-specific promoter of cancer relapse (Fig. 9g).

## Discussion

YAP has the interesting property of promoting cancer and expanding undifferentiated stem/progenitor cells. Our study provides the first demonstration that YAP promotes the transcription of a subset of genes enriched in MaSCs specifically in BLBC and further identifies a novel SRF–YAP–IL6 signalling pathway in the promotion of MaSC-like features in basal cells and BLBC.

Kupperwasser and colleagues reported that TAZ induces luminal cells to adopt basal cell characteristics^39^. Our study has shown that YAP or TAZ can induce MaSC signature-gene expression and MaSC-like properties in MCF-10A and 4T1 cells, which are already basal cells. Therefore, while TAZ may be specifically involved in luminal-basal cell plasticity, YAP and TAZ may redundantly be involved in promoting MaSC-like properties in basal cells. Thus, our data and those of others suggest that YAP and TAZ are broadly involved in mammary gland differentiation from MaSCs to luminal cells. Consistently, a recent report by Pan and colleagues demonstrated that hyperactivation of YAP in mouse mammary glands causes defects in terminal differentiation^40^.

Although we have shown that SRF and YAP are necessary for induction of MaSC-like properties in mammary epithelial cells and breast cancer cell lines, it should be noted that high expression of these two proteins is not sufficient to fully induce MaSC-like properties. For example, our data showed that SRF and YAP co-overexpression is not sufficient to induce MaSC-like properties in luminal-type cancer cell lines. We believe that, in addition to simple coexpression of SRF and YAP, other activation cues may be required for full-fledged MaSC-like properties. One such example could be EMT-mediated activation of YAP. In fact, we showed greater YAP nuclear localization in more MaSC-like basal B-type breast cancers (Supplementary Fig. 12). This might explain why the claudin-low breast cancer subtype, which is the most MaSC-like, did not have highest YAP/TAZ mRNA levels. The mesenchymal phenotype of claudin-low subtype cancer may achieve its MaSC-like properties through post-transcriptionally activated YAP. It will thus be important to elucidate the mechanisms by which these cellular contexts cooperate with SRF–YAP to induce MaSC-like properties.

A notable finding is that the well-characterized oncogenic pathway downstream of YAP involving TEAD and CTGF are dispensable for YAP induction of MaSC-like properties. We suggest that both TEAD and SRF are required for YAP's transforming activity; however, the various cellular phenotypes conferred by YAP can be mechanistically separable. For example, TEAD is mainly involved in YAP-induced EMT and cell proliferation, and SRF is mainly involved in the induction of stemness properties in mammary epithelial cells. A genome-wide study designed to classify YAP transcriptional targets according to their dependence on certain transcription factors could shed light on the role of various transcription factor partners in the oncogenic function of YAP. We found that TEAD interferes with YAP induction of MaSC-like properties rather than supporting it. We believe that TEAD is unlikely to be directly involved in IL6 expression for three reasons. One is that TEAD deficient in binding YAP was still able to suppress IL6 expression as efficiently as wild-type TEAD. Second, we also demonstrated YAP and SRF interaction without presence of TEAD. Lastly, the EMSA assay showed that presence of exogenous TEAD does not alter DNA mobility shift caused by SRF and YAP. All these lines of evidence point to the notion that TEAD is unlikely to have a functional role as a part of the SRF–YAP complex. Future studies should reveal the exact mechanism by which TEAD suppresses stemness in mammary epithelial cells. Finally, verteporfin, a chemical inhibitor of YAP-TEAD interaction^41^, has been shown to be a candidate therapeutic agent for YAP-overexpressing cancers. However, our results indicate that inactivating TEAD tended to increase MaSC-like properties instead of decreasing them and was largely ineffective in reducing stemness in Ras-transformed cancer cells. Therefore, caution needs to be warranted in the use of verteporfin for YAP-activated cancers in certain contexts.

YAP is a critical downstream effector molecule for the Hippo tumour-suppressor pathway^10^. Accordingly, loss of the Hippo pathway mimics the phenotype of YAP overexpression, manifesting as expansion of stem/progenitor cells in various organs. We have previously demonstrated that YAP is involved in oval cell-specific proliferation in the liver^6^. Our current finding that YAP confers stemness to epithelial cells in the basal layer but not to those in luminal layers suggests that YAP preferentially confers stemness in specific tissue compartments. Our finding raises the intriguing possibility that deregulation of IL6 by hyperactive YAP may be responsible for expansion of stem/progenitor cells in Hippo pathway-knockout mice. Mice with conditional knockout of the Hippo pathway might shed light on the importance of SRF and IL6 in the expansion of stem/progenitor cells in various organs such as mammary gland, liver and intestine, where Hippo pathway-dependent restriction of adult stem/progenitor cells is found. These future studies will clarify the specific role of the Hippo-YAP pathway and downstream signals involving IL6 and SRF in progenitor cells *in vivo*. The concept of a SRF–IL6 as a critical signalling module for YAP's stemness-promoting activity can similarly be applied to cancer. In fact, IL6-knockout mice show resistance to various tumorigenic insults to the liver, skin and intestine^42^,^43^,^44^. Moreover, SRF may be involved in cancer metastasis^17^. In light of a recent report that Unpaired is a critical non-cell-autonomous regulator of cancer invasion^45^, future studies of SRF–IL6 in tumours generated by deficiencies in the Hippo signalling pathway should delineate the universally conserved mechanisms downstream of Hippo signalling in tissue homeostasis.

SRF and YAP exhibit shared activation mechanisms, with both proteins being activated by common mechanical and chemical cues. We speculate that the SRF–YAP complex may be responsible for responding to extracellular cues for changes in cellular physiology such as stemness and invasiveness. Notably, signals that activate SRF–YAP are engaged virtually immediately on tissue injury, such as in dextran sulfate-mediated colitis^46^. Therefore, these upstream signals might activate SRF and YAP during tissue injury under normal, homeostatic conditions, thereby inducing regenerative responses. Deregulation of SRF and YAP could cause constitutively injured, hyper-regenerative responses with excessive cell proliferation, invasion and expansion of stem/progenitor cells. Additional studies will be necessary to establish whether upstream signals that regulate SRF and YAP act in parallel or exhibit crosstalk.

## Methods

### Cell culture

293T and MDA-MB-231, MCF-7 cells were cultured in DMEM supplemented with 10% fetal bovine serum (FBS). Other breast cancer cell lines (HCC1954, MDA-MB361, SKBR3, MDA-MB453, T47D, ZR-75-1, BT-20, HCC38 and Hs578T) were cultured in RPMI-1640 medium supplemented with 10% FBS. 293T, MCF-7 and MDA-MB-231 cells are obtained from American Type Culture Collection, and other breast cancer cell lines were obtained from Korea Research Institute of Bioscience and Biotechnology. MCF-10A cells were cultured as described in ref. 47. Cell lines were validated by DNA fingerprinting at TPOX, TH01, vWA and D5S818 loci. Cells were routinely tested for presence of mycoplasma with 4,6-diamidino-2-phenylindole staining.

### Flow cytometry

Cells were seeded in six-well plates at 10^5^ cells per well. On the next day, cells were trypsinized and stained with anti-CD44-PerCP-Cy5.5 (eBioScience, 1:100) and anti-CD24-biotin (eBioScience, 1:100) antibodies, and then subsequently stained with fluorescein isothiocyanate-conjugated streptavidin (BD Biosciences, 1:200). Stained cells were analysed using a FACSCalibur flow cytometry system (BD Biosciences), and fluorescence-activated cell sorting (FACS) plots were analysed with the FlowJo software (Tree Star Inc.).

### Mammosphere assay

Cells were trypsinized, and 10^4^ cells per well in 2.5 ml of DMEM/F12 supplemented with B-27 (Gibco), 20 ng ml^−1^ human epidermal growth factor (Peprotech), 20 ng ml^−1^ basic fibroblast growth factor (KOMA Biotech) and 4 μg ml^−1^ heparin (Sigma) were seeded in six-well plates pre-coated with poly-HEMA (Sigma). Mammospheres were counted 5–10 days later.

### Microarray analysis

RNA was collected and analysed using a Human HT-12v4 expression chip (Illumina). All expression values (in log2) were normalized and statistically analysed to identify genes whose average expression at 2 and 6 h after the 4-OHT treatment was significantly upregulated (*P*<0.001) in MCF-10A ER^T2^-YAP 2SA cells without being significantly changed (*P*>0.01) in control MCF-10A ER^T2^ cells. Heatmaps for gene sets of interest were generated using Multi-experiment Viewer. The raw data from microarray analyses are available in Gene Expression Omnibus (GEO, GSE60579).

### ChIP assay

DNA in cells from two confluent 100-mm culture dishes (∼2 × 10^7^ cells total) was pretreated with 1.5 mM ethylene glycol bis(succinimidylsuccinate) (Sigma) for 30 min at room temperature to capture proteins indirectly bound to DNA, and then crosslinked by incubating with 1% formaldehyde for 15 min. After DNA crosslinking, cells were sonicated by Bioruptor (BMS Co.) in SDS lysis buffer (50 mM Tris-Cl pH 8.0, 1% SDS and 10 mM EDTA) and diluted 10-fold with dilution buffer (16.7 mM Tris-Cl pH 8.0, 167 mM NaCl, 1.1% Triton X-100 and 1.2 mM EDTA) and processed for ChIP assays using 2 μg of anti-YAP antibody (H-125, Santa Cruz Biotechnology), anti-TEAD4 antibody (Abcam) or anti-SRF antibody (Cell Signaling) and Protein A/G agarose (GenDEPOT). YAP 5SA is used for maximal efficiency in YAP binding to the chromatin. See Supplementary Table 1 for primers used.

### GEO microarray analysis

Microarray data for independent sets of breast cancer cohorts were downloaded from NCBI GEO. Microarray data were normalized and analysed using geWorkbench. The microarray cohorts used for this study are GSE1456 (ref. 48), GSE3494 (ref. 49), GSE21653 (ref. 50) and GSE31448 (ref. 51).

### Tissue array analysis

Tissue arrays and corresponding information, including tumour subtypes and grade, were obtained from patients with informed consent in Yonsei Severance Hospital. Immunohistochemistry was performed using the following antibodies: anti-YAP (Cell Signaling, 1:200), anti-TAZ (V386; Cell Signaling, 1:200), anti-SRF (Cell Signaling, 1:200) and anti-ALDH1A (ab52492; Abcam, 1:200). The degree of antigen staining was evaluated on a scale of 1–4, with 1 and 2 designated as low, and 3 and 4 designated as high. This study was approved by the Institutional Review Board of Yonsei University Severance Hospital.

### *In vivo* mouse experiments

Mouse experiments were performed in accordance with procedures approved by the Korea Advanced Institute of Science and Technology-Animal Care and Use Committee.

### Xenograft assay

A 1:1 mixture of Matrigel and PBS containing 10^6^ or 4 × 10^6^ cells in a total volume of 100 μl was injected subcutaneously into 6–8-week-old female nude mice. The presence of palpable tumours was examined 4 weeks after xenograft. The number of tumour-initiating cells was quantified using the ELDA (Extreme Limiting Dilution Analysis) software^52^.

### Syngeneic graft assay

For 4T1 tumour immunohistochemistry, 10^5^ cells in 100 μl of a 1:1 mixture of Matrigel and PBS were injected into the fourth inguinal mammary fat pad of 5–6-week-old female BALB/c mice. Tumours were grown for 15 days and excised for immunohistochemical analysis. For limiting dilution assays, 10 or 50 4T1 cells were injected as described for immunohistochemical analyses and analysed similarly to xenograft assays 15 days after injection. The number of tumour-initiating cells was quantified using the ELDA software.

### Co-immunoprecipitation

Cells were washed twice with PBS and then treated with the crosslinking agent dithiobis(succinimidyl propionate) (1 mM; Pierce) for 2 h at 4°C. Cells were then lysed in binding buffer (20 mM Tris-Cl pH 8.0, 100 mM NaCl, 1mM MgCl_2_, 0.5% NP-40). Cleared lysate was incubated with protein S agarose (Novagen) for 1 h to immunoprecipitate S-tagged proteins, and with anti-Flag or anti-SRF antibody (Cell Signaling) overnight and protein A/G agarose (Genedepot Inc.) for 1 h to immunoprecipitate Flag-tagged proteins and endogenous SRF, respectively. One hundred units of benzonase (Enzynomics) were added to remove contaminating DNA in cell extracts. Lysates were washed five times with binding buffer, boiled in Laemmli sample buffer and subjected to SDS–PAGE. Uncropped, original images of the western blot analyses are shown in Supplementary Fig. 14.

### *In vitro* pulldown assay

Glutathione *S*-transferase (GST)-tagged YAP (51–394) protein was purified from *Escherichia coli*. One milligram of 293T extract expressing S-tag-Flag-SRF was first incubated with protein S agarose (Novagen) for 1 h and washed five times with binding buffer supplemented with 500 mM LiCl to maximally eliminate binding proteins. Then, the bead with S-tag-Flag-SRF was resuspended with binding buffer and incubated with 50 μg of GST protein and 100 units of benzonase overnight at 4 °C. Then, the bead was washed with binding buffer five times, boiled in Laemmli sample buffer and subjected to SDS–PAGE. Uncropped, original image of the western blot analyses are in Supplementary Fig. 14.

### EMSA

PCR products generated using ChIP assay primers targeting the *IL6* promoter CArG locus were labelled with T4 polynucleotide kinase. Labelled probe (10 fmol) was incubated for 2 h at 4 °C with 2 μg of MDA-MB-231 nuclear extract and 1 μg of GST-tagged YAP (51–394) protein (used for *in vitro* pulldown assays) in EMSA buffer (15 mM HEPES-KOH pH 7.9, 42 mM NaCl, 2 mM MgCl_2_, 0.1 μg ml^−1^ bovine serum albumin (BSA), 0.5 mM dithiothreitol, 5% glycerol, 1 mM EDTA and 50 μg ml^−1^ poly[I:C]). Unlabelled competitor oligonucleotide designed to encompass 20 base pairs that include the *IL6* promoter CArG box (5′- AGCTTCCTTACAAGGAAAAC -3′) or its mutant derivative (5′- AGCTTAATTACAAGGAAAAC -3′) was added at 1,000-fold molar excess (10 pmol) to the labelled probe. Supershift was performed by adding 1 μg of control, nonspecific IgG or haemagglutinin (HA) antibody. The DNA–protein complex was then resolved on a 5% polyacrylamide gel in TBE and, after drying, was analysed using autoradiography. Uncropped, original image of the autoradiography is in Supplementary Fig. 14.

### F/G-actin fractionation

F/G-actin fractionation was performed as described in ref. 53. Briefly, cells were lysed with G-actin extraction buffer (1% Triton X-100, 10 mM Tris-Cl pH 7.5, 1 mM EGTA, 50 mM NaCl and 15% glycerol) at room temperature; 5% of the lysate was kept as total actin. After ultracentrifugation at 150,000g for 1 h at room temperature, 5% of the supernatant was kept as the G-actin fraction. The pellet was then lysed with F-actin extraction buffer (4 M urea, 1% SDS, 0.5% Triton X-100, 5 mM Tris-Cl pH 7.5, 0.5 mM EGTA, 25 mM NaCl, 15% glycerol and 50 mM dithiothreitol) with rigorous vortexing; 20% of the supernatant was kept as the F-actin fraction. All fractions were then boiled in Laemmli sample buffer and subjected to SDS–PAGE. Uncropped, original image of the western blot analyses are in Supplementary Fig. 14.

### Immunofluorescence

Cells seeded on gelatin-coated coverslips were fixed with 4% paraformaldehyde, permeabilized with 0.1% Triton X-100 in PBS and blocked with 1% BSA in PBS. Cells were then incubated with primary antibody overnight at 4 °C. After washing with PBS, cells were incubated with secondary antibody for 1 h at 37 °C, and then washed with PBS and mounted with Vectashield (Vector Laboratories).

### Antibodies

The following antibodies were used (dilutions are for western blot applications unless otherwise indicated): anti-Flag (Wako; 1:1,000), anti-HA (Covance; 1:10,000), anti-YAP (raised against the C-terminal human YAP antigen; 1:2,000), TAZ (Cell Signaling (V386); 1:1,000 for western blotting, 1:200 for immunohistochemistry), anti-β-actin (Sigma; 1:10,000), anti-SRF (Cell Signaling (D71A9); 1:1,000 for western blotting, 1:200 for immunohistochemistry), anti-CTGF (Santa Cruz; 1:250), anti-E-cadherin (BD Biosciences; 1:2,000), anti-N-cadherin (BD Biosciences; 1:500), anti-ALDH1A1 (Abcam (EP1933Y); 1:100 for immunohistochemistry), anti-IL6 (Abcam; 1:500 for immunohistochemistry) and anti-MRTFB (Bethyl Laboratories; 1:100 for immunofluorescence).

### shRNA

shRNA target sequences used for this study are as follows: shYAP, 5′- CAGGTGATACTATCAACCAAA -3′; shTAZ, 5′- AGGTACTTCCTCAATCACA -3′; shSRF #1, 5′- CGATGTTTGCCATGAGTATTA -3′; shSRF #2, 5′- GTGAGACAGGCCATGTGTATA -3′; shIL6 #1, 5′- GAACTTATGTTGTTCTCTA -3′; shIL6 #2, 5′- AGAACGAATTGACAAACAA -3′; shCTGF #1, 5′- AAATCTCCAAGCCTATCAAGT -3′; shCTGF #2, 5′- CTGCACCAGCATGAAGACATA -3′; shTCF3, 5′- CCCAGCAGCCTCTCTTCATCC -3′; shSlug #1, 5′- CCCATTCTGATGTAAAGAAAT -3′; shSlug #2, 5′- GAGTGACGCAATCAATGTTTA -3′; shMRTFA, 5′- GACTATCTCAAACGGAAGATT -3′; shMRTFB, 5′- GCAGACACTTTCACCGAGATT -3′ shSrf (mouse), 5′- GCCAGCATTCACAGTCACCAA -3′; shIl6 (mouse), 5′- CAATGGCAATTCTGATTGTA -3′.

### RT–PCR

Cells were lysed in Ribo-Ex (GeneAll Inc.) solution, and RNA was purified according to the manufacturer's instructions. cDNA was synthesized from total RNA using MMLV reverse transcriptase (Enzynomics) by incubating for 2 h at 37 °C. All gene expression levels were normalized to those of hypoxanthine phosphoribosyltransferase 1 in the case of human cells, and to that of glyceraldehyde-3-phosphate dehydrogenase in the case of mouse cells. See Supplementary Table 1 for primers used.

### Statistics and data processing

All statistical analyses, including two-tailed *t*-tests and log-rank tests for survival analyses, were performed using Graphpad Prism software. Analyses were performed with two-tailed *t*-tests, unless otherwise indicated. Sample exclusion was never carried out and appropriate sample size was defined to reveal significant difference we observed. No randomization or blinding was performed.

## Additional information

**How to cite this article:** Kim, T. *et al*. A basal-like breast cancer-specific role for SRF–IL6 in YAP-induced cancer stemness. *Nat. Commun.* 6:10186 doi: 10.1038/ncomms10186 (2015).

## Supplementary Material

Supplementary Information Supplementary Figures 1-14, Supplementary Table 1 and Supplementary Reference

Supplementary Data 1 List of YAP induced target genes described in Figure 1b List of 226 genes whose expressions were significantly (p0.01) in control MCF-10A ERT2. Average fold change and corresponding p values are listed for both control and experimental sets

Supplementary Data 2 Gene Set Enrichment Analysis results for MaSC signature-gene expression in MCF-10A ERT2-YAP 2SA cells after treatment with 4-OHT Transcription factor signatures and corresponding enrichment scores, p values and False Discovery Rate (FDR) q values are listed.
