# Supplementary material for: A basal-like breast cancer-specific role for SRF–IL6 in YAP-induced cancer stemness
Source: Nat Commun. 2015 Dec 16;6:10186. doi: 10.1038/ncomms10186 (PMC4703869; doi:10.1038/ncomms10186)
Supplement: Supplementary Information — Supplementary Figures 1-14, Supplementary Table 1 and Supplementary Reference [file ncomms10186-s1.pdf]

Supplementary Figures

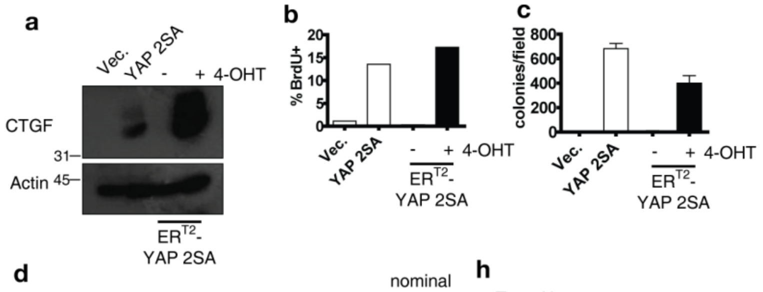

d

Cordenonsi_YAP_Conserved_Signature	nominal p-value
ERYAP-EtOH vs. ER-EtOH	0.321
ERYAP-EtOH vs. ERYAP-4-OHT_2Hrs	<0.001
ERYAP-EtOH vs. ERYAP-4-OHT_6Hrs	<0.001

h

Term Name	p-value
thoracic cancer	2.00e-9
malignant neoplasm of breast	5.40e-9
mammary cancer	5.42e-9
malignant neoplasm of thorax	5.65e-9
reproductive system cancer	6.78e-8
gastrointestinal neoplasm	3.54e-7
digestive system cancer	3.72e-7
lung disease	1.34e-6
diabetic angiopathy	1.95e-6
lower respiratory tract disease	1.96e-6

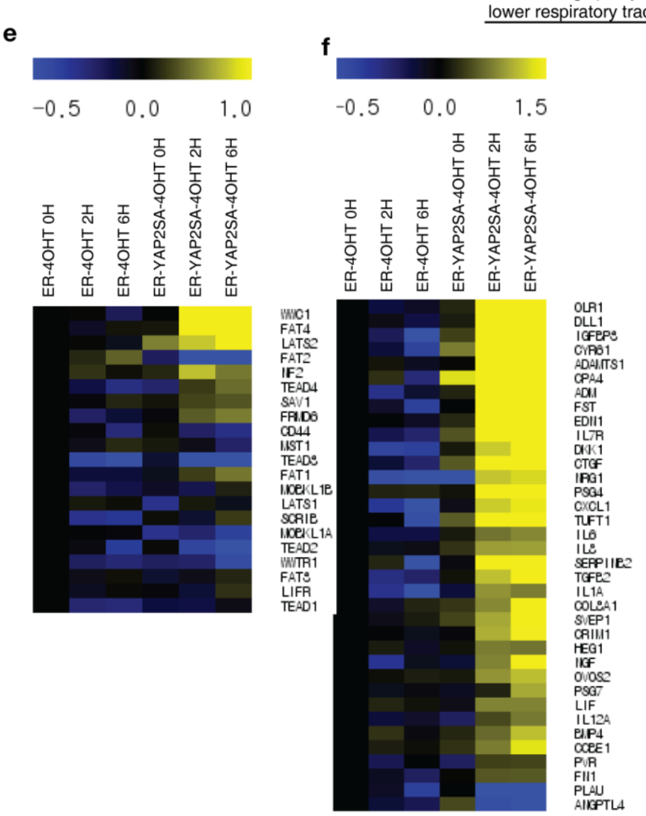

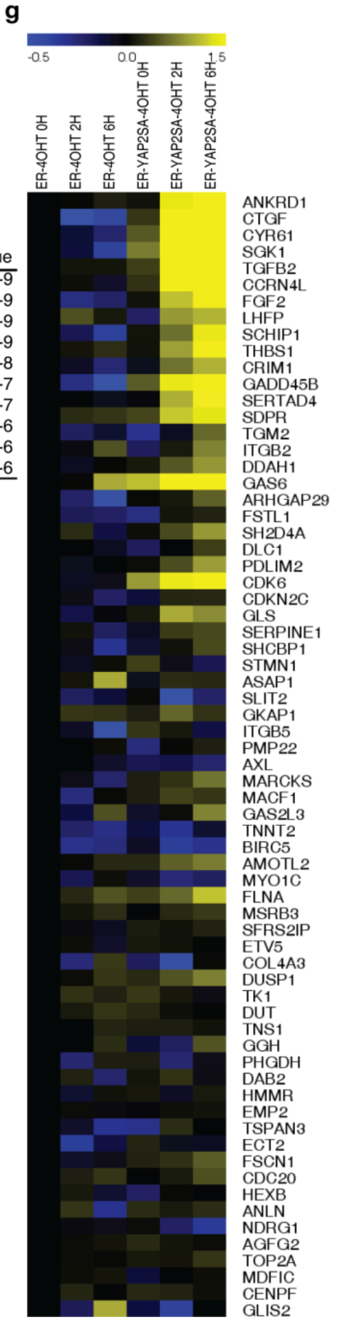

Supplementary Figure 1. Summary of microarray analysis of MCF-10A ERT2-YAP 2SA cells. (a–d) Validation of the ER^{T2}-YAP 2SA system. MCF-10A cells were infected with the indicated retroviruses. (a) Western blot, (b) BrdU assay after 48-hour serum starvation (n=1 experiment), and (c) soft agar colony counts for cells generated (n=3 experiments). (d) Gene set enrichment analysis summary for conserved YAP target genes. (e) Heatmap analysis of selected Hippo pathway genes. (f) Heatmap analysis of selected secreted genes. (g) Heatmap analysis of YAP/TAZ signature genes listed by Piccolo and colleagues[1]. (h) Gene Ontology

analysis of genes significantly upregulated following YAP induction. Data are presented as means ± s.e.m..

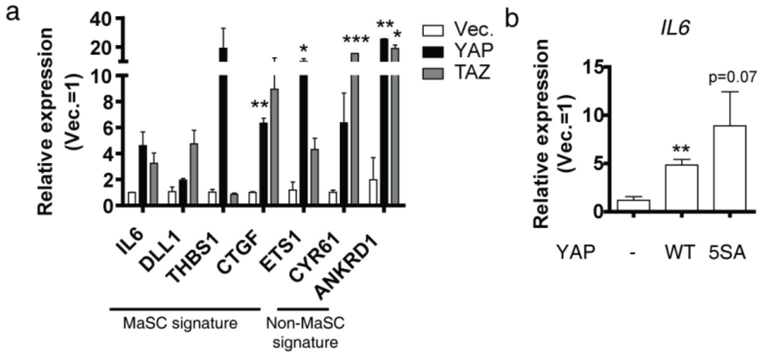

Supplementary Figure 2. YAP and TAZ are redundant with respect to MaSC signature gene expression. (a) qRT-PCR analyses of the expression of the indicated genes in MCF-10A cells overexpressing YAP or TAZ (n=2 replicates). (b) qRT-PCR data for IL6 expression in MCF-10A cells expressing indicated forms of YAP (n=2 experiments with 2 replicates). Data are presented as means ± s.e.m. (*P<0.05, **P<0.01, ***P<0.001, student's t-test used in all analyses).

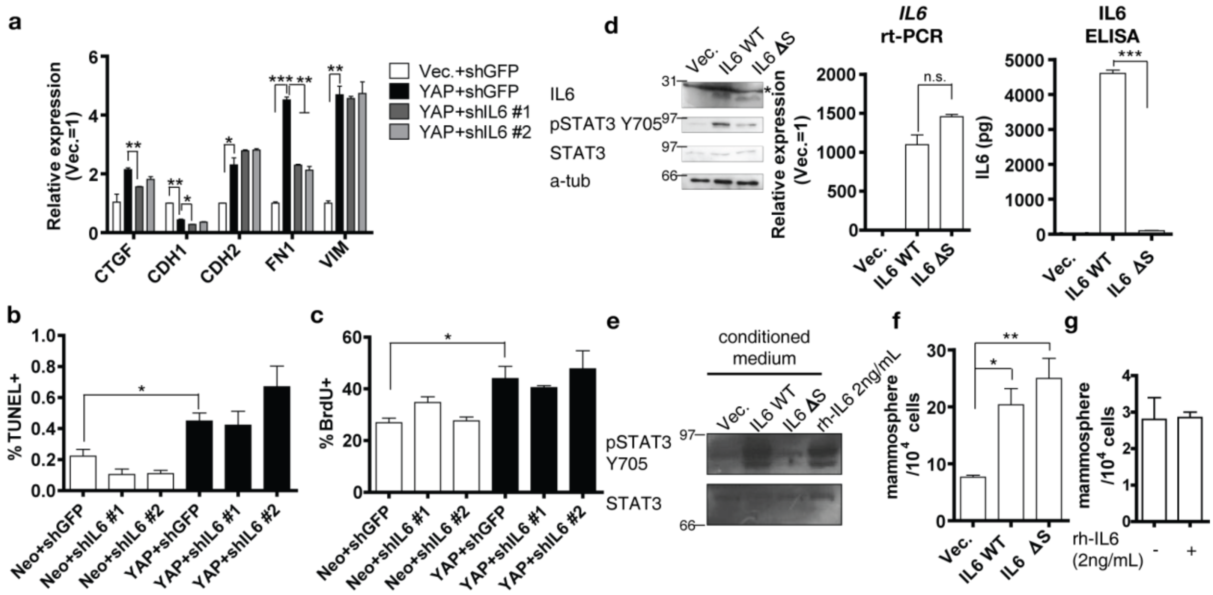

Supplementary Figure 3. Intracellular IL6 signaling promotes MaSC-like properties. (a) qRT-PCR analyses of the expression of the indicated genes in MCF-10A cells infected with the indicated viruses (n=2 replicates). (b) TUNEL assay (n=3 experiments) and (c) BrdU uptake assay (n=3 experiments) for MCF-10A cells infected with the indicated viruses. (d-f) MCF-10A cells were infected with retrovirus overexpressing either wild-type IL6 (IL6 WT) or signaling peptide-deficient IL6 (IL6 ΔS). (d) Western blot, qRT-PCR (n=2 replicates) and ELISA (n=3 replicates) analyses of MCF-10A cells expressing indicated forms of IL6 for

detection of intracellular IL6, *IL6* mRNA and secreted IL6, respectively. Asterisk in western blot indicates a non specific band. (e) Western blot analysis of serum-starved MCF-10A cells treated with recombinant IL6 (rh-IL6) or conditioned medium from the indicated cells. (f) Mammosphere assay of MCF-10A cells expressing the indicated genes (n=3 experiments). (g) Mammosphere assay of MCF-10A cells treated with indicated amount of recombinant human IL6 (rhIL6) (n=3 experiments). Data are presented as means ± s.e.m. (*P<0.05, **P<0.01, ***P<0.001, n.s., not significant [P>0.05], student's t-test used in all analyses).

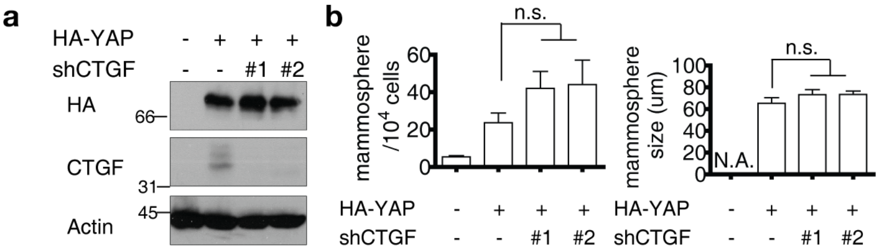

Supplementary Figure 4. CTGF is dispensable for YAP induction of MaSC properties. (a) Western blot analysis of MCF-10A cells transduced with the indicated viruses. (b) Number and sizes of mammospheres formed by cells generated in (a) (n=3 experiments for frequency, n>37 for size of mammospheres). Data are presented as means ± s.e.m. (n.s., not significant, student's t-test used in all analyses).

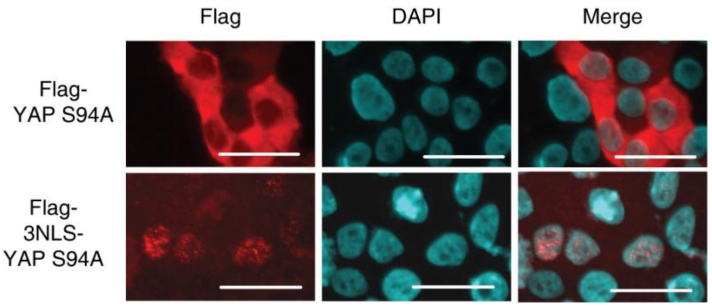

Supplementary Figure 5. Localization of NLS-tagged YAP mutant protein. MCF-10A cells expressing the indicated genes were analyzed by immunofluorescence using an anti-Flag antibody. Scale bar: 20μm.

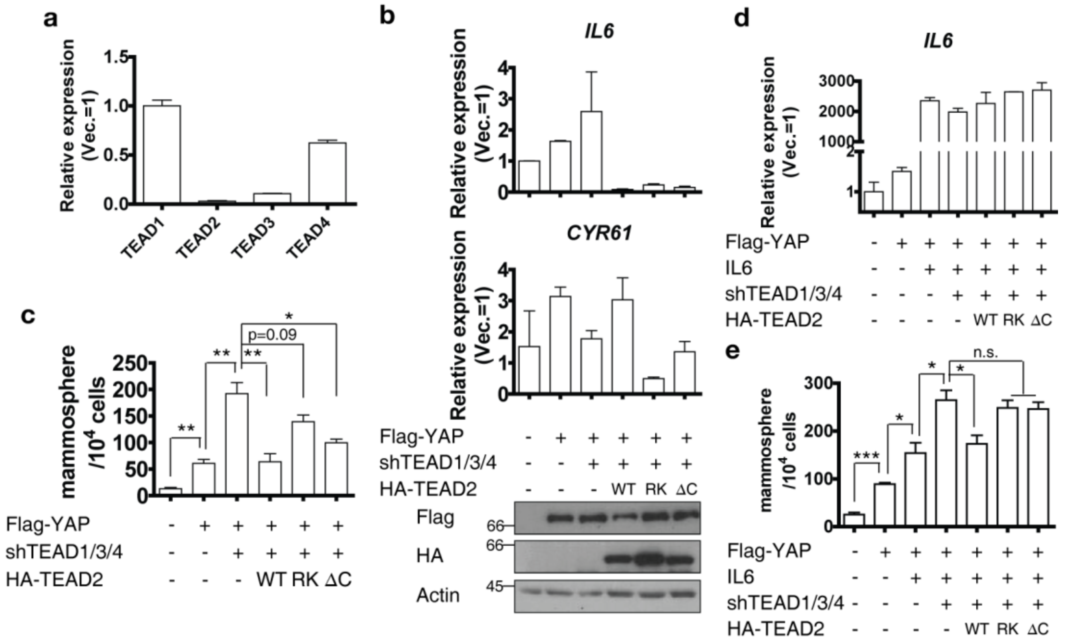

Supplementary Figure 6. TEAD is dispensable for YAP induction of MaSC properties. (a) MCF-10A cells were analyzed for TEAD expression by qPCR (n=2 replicates). (b,c) MCF-10A cells expressing YAP were infected with lentivirus expressing TEAD1/3/4 shRNA together with wild-type (WT), DNA-binding–deficient (R95K) or YAP-binding–deficient TEAD2 (ΔC). (b) Western blotting and qRT-PCR (n=2 replicates). (c) Statistical analyses of the number of mammospheres formed by MCF-10A cells expressing the indicated viruses (n=3 experiments). (d,e) MCF-10A cells were infected with the indicated viruses. (d) qRT-PCR analysis of IL6 expression (n=2 replicates). (e) Statistical analyses of the number of mammospheres formed by MCF-10A cells expressing the indicated viruses (n=3 experiments). Data are presented as means ± s.e.m. (*P<0.05, **P<0.01, ***P<0.001; n.s., not significant [P>0.05] , student's t-test used in all analyses).

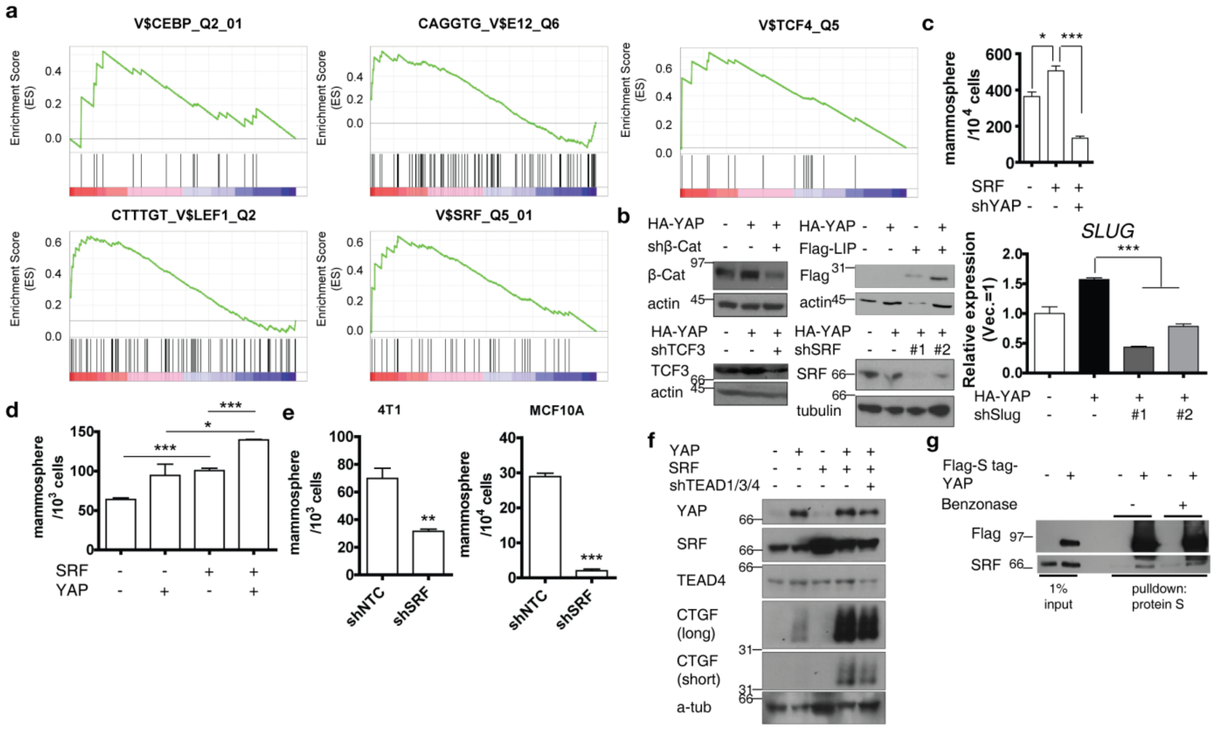

Supplementary Figure 7. SRF and YAP induce MaSC-like properties. (a) Representative graph of the GSEA analysis presented in Figure 3a. (b) Confirmation of successful inactivation of transcription factors in Figure 3b by Western blot and qRT-PCR analyses (n=2 replicates) of the indicated genes. (c,d) Statistical analyses of the number of mammospheres formed by 4T1 cells expressing the indicated viruses (n=3 experiments). (e) Mammosphere forming frequency for 4T1 and MCF-10A cells depleted of SRF. (f) Western blot analyses of MCF-10A cells expressing the indicated viruses. (g) Co-immunoprecipitation analysis of DSP crosslinked 293T cells expressing S-tagged YAP and pretreated with benzonase. Data are presented as means ± s.e.m. (*P<0.05, ***P<0.001, student's t-test used in all analyses).

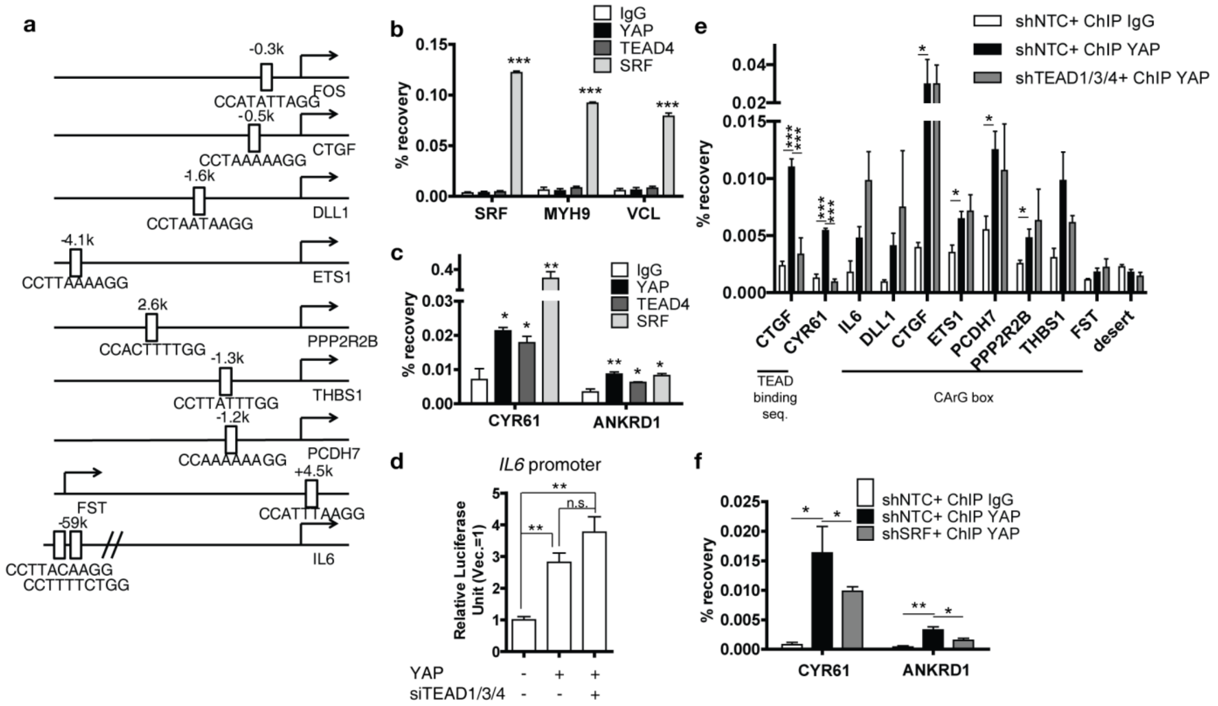

Supplementary Figure 8. YAP interacts with SRF to induce MaSC-like properties. (a) CArG boxes in the promoters of MaSC signature genes examined. (b) ChIP analyses of the indicated SRF-MRTF–dependent gene promoters with DNA used in Figure 4f (n=3 experiments). (c) ChIP analyses of the indicated YAP targets uninfluenced by SRF depletion with DNA used in Figure 4f (n=3 experiments). (d) Luciferase assays of the *IL6* promoter CArG box in 293T cells overexpressing the indicated genes (n=3 experiments). (e) ChIP analyses of the indicated MaSC signature genes (n=3 experiments). (f) ChIP analyses of YAP targets not influenced by SRF depletion with DNA used in Figure 4i (n=3 experiments). Data are presented as means ± s.e.m. (*P<0.05, **P<0.01, ***P<0.001, n.s., not significant [P>0.05] , student's t-test used in all analyses).

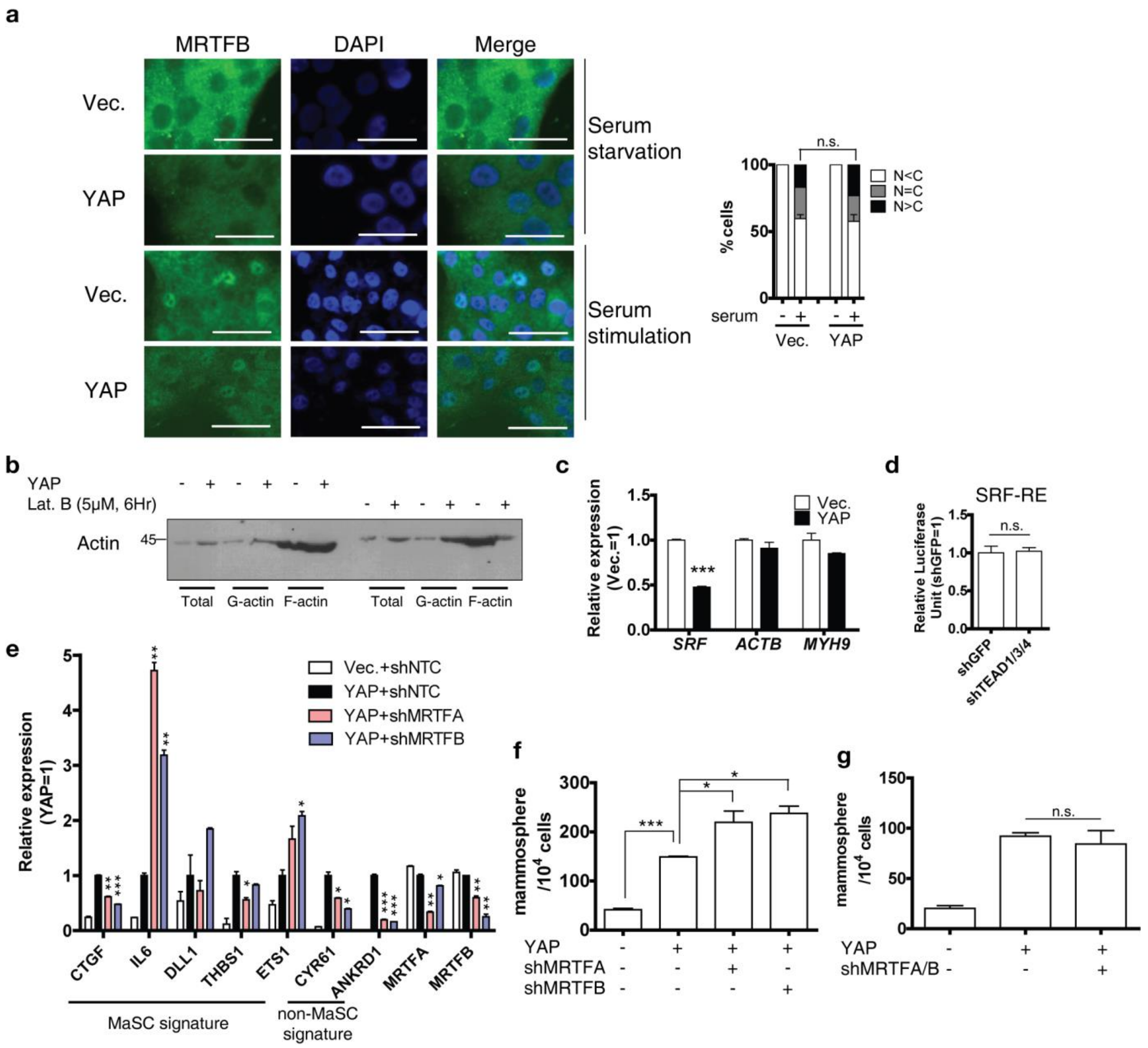

Supplementary Figure 9. YAP does not influence SRF-MRTF activity. (a) Control and Yap-overexpressing MCF-10A cells were either serum starved or stimulated and analyzed for MRTFB localization by immunofluorescence assay. Bar graph on the right shows quantification (n=3 experiments). Scale bar: 20μm. (b) F/G-actin fractionation assay of MCF-10A cells overexpressing YAP or treated with Latrunculin B. (c) qRT-PCR analyses of SRF-MRTF target genes in MCF-10A cells overexpressing YAP (n=2 replicates). (d) Luciferase assays of the SRF-MRTF-responsive SRF-RE promoter in 293T cells treated with the indicated shRNA (n=3 experiments). (e) qRT-PCR analyses of YAP target genes in MCF-10A cells infected with the indicated viruses (n=2 replicates). (f) Quantification of mammosphere assay and statistical analysis of cells generated in (e) (n=3 experiments). Data are presented as means ± s.e.m. (*P<0.05, **P<0.01, ***P<0.001, student's t-test used in all analyses).

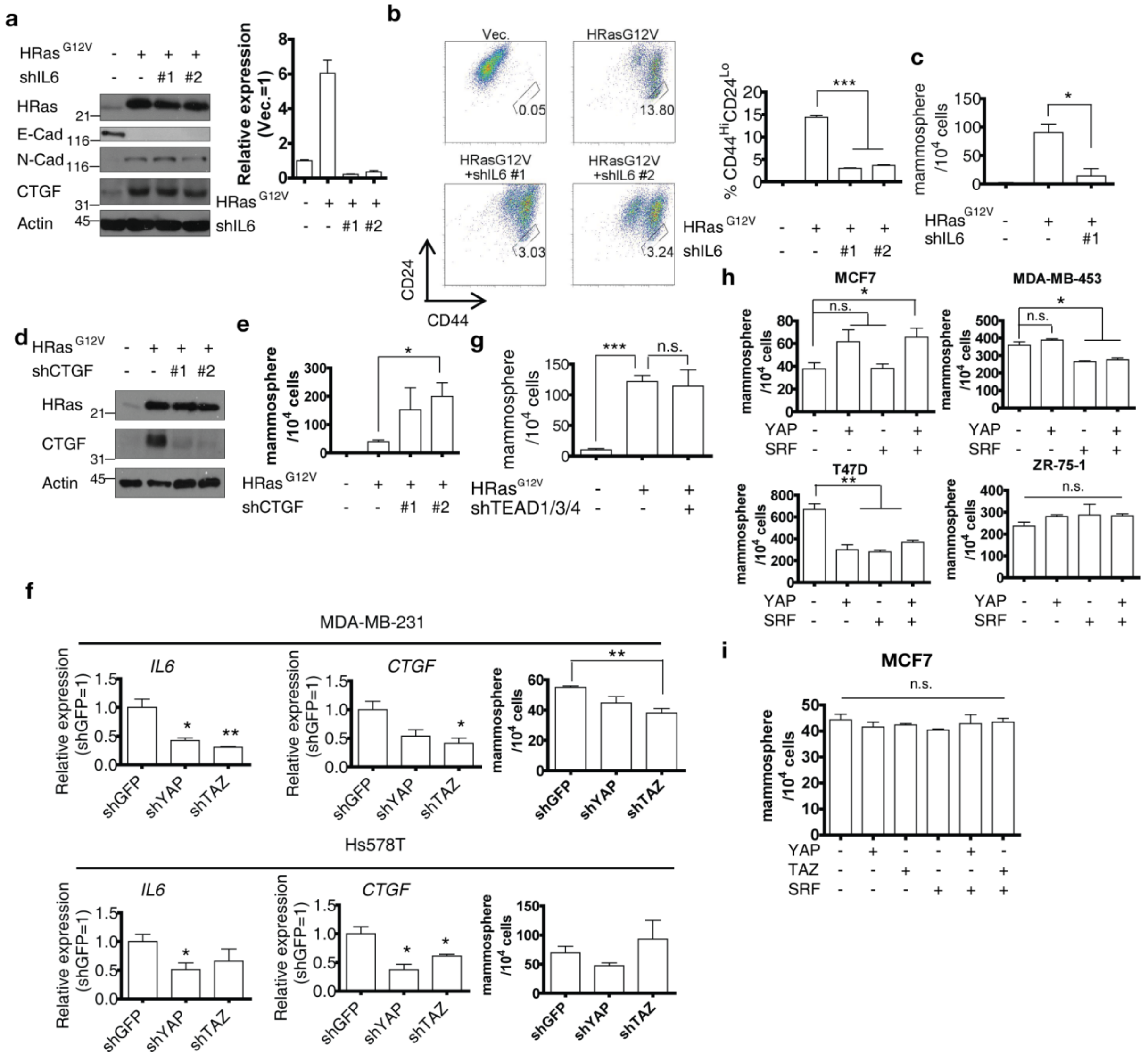

Supplementary Figure 10. SRF-YAP promotes Ras-induced cancer stem cell formation through regulation of IL6. (a-c) MCF-10A cells were transformed with H-Ras^{G12V} and then infected with shRNA against IL6. (a) Western blot and qRT-PCR analyses of generated cells (n=2 replicates). (b) Representative FACS plot and corresponding statistical analysis (n=3 experiments), and (c) quantification of mammosphere assays performed using cells generated in (a) (n=3 experiments). (d, e) MCF-10A cells were transformed with H-Ras^{G12V} and then infected with shRNA against CTGF. (d) Western blot analysis of generated cells. (e) Quantification of mammosphere assays performed using cells generated in (d) (n=3 experiments). (f) qRT-PCR and statistical analyses of mammosphere assays in cells depleted of YAP or TAZ. (g) Quantification of mammosphere assays performed using cells infected with virus expressing HRas^{G12V} and shRNA against TEAD (n=3 experiments). (h) Quantification of mammosphere assays performed using luminal-type breast cancer cell lines overexpressing SRF and/or YAP (n=3 experiments). (i) Quantification of mammosphere assays performed using MCF7 cells overexpressing SRF and/or YAP/TAZ (n=3 experiments). Data are presented as means ±

s.e.m. (*P<0.05, **P<0.01, ***P<0.001; n.s., not significant [P>0.05] , student's t-test used in all analyses).

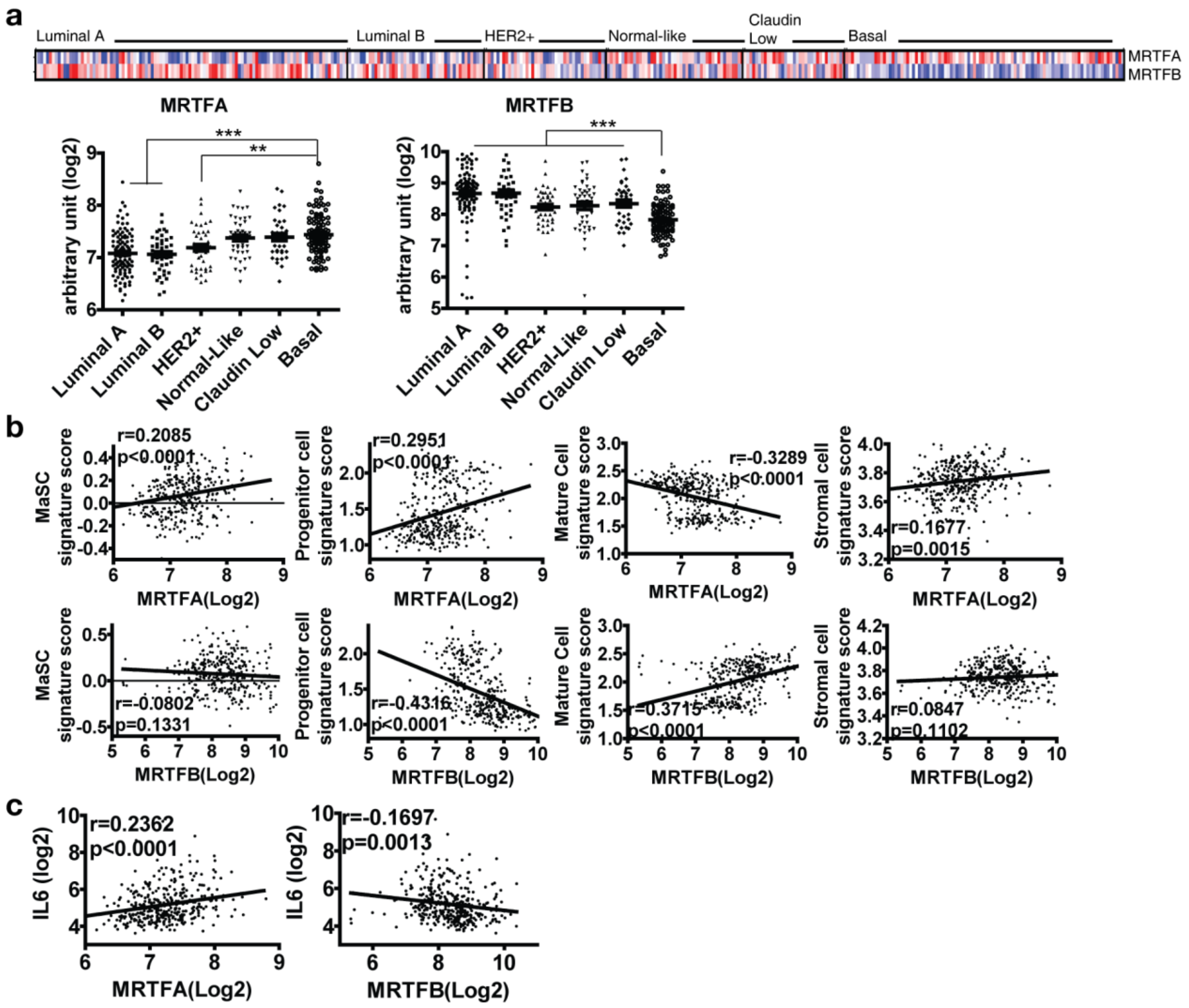

Supplementary Figure 11. MRTF family gene expression in breast cancer. (a) Heatmap and statistical analyses of MRTFA and MRTFB expression in a panel of breast cancers (GSE31448; n>27for each subtype) (b) Scatter plot of signature scores for the indicated cell types against MRTFA or MRTFB expression levels (n=357; correlation tested using Pearson's correlation coefficient, r). (c) Scatter plot of IL6 and CTGF expression against MRTFA or MRTFB expression levels (n=357; correlation tested using Pearson's correlation coefficient, r). Data are presented as means ± s.e.m. (**P<0.01, ***P<0.001).

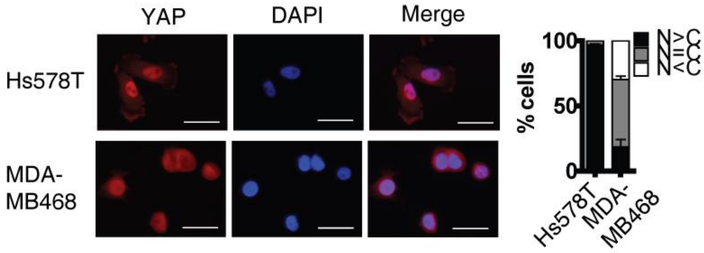

Supplementary Figure 12. Immunofluorescence analysis and statistical quantification of YAP expression in the indicated cell lines. Scale bar: 20μm.

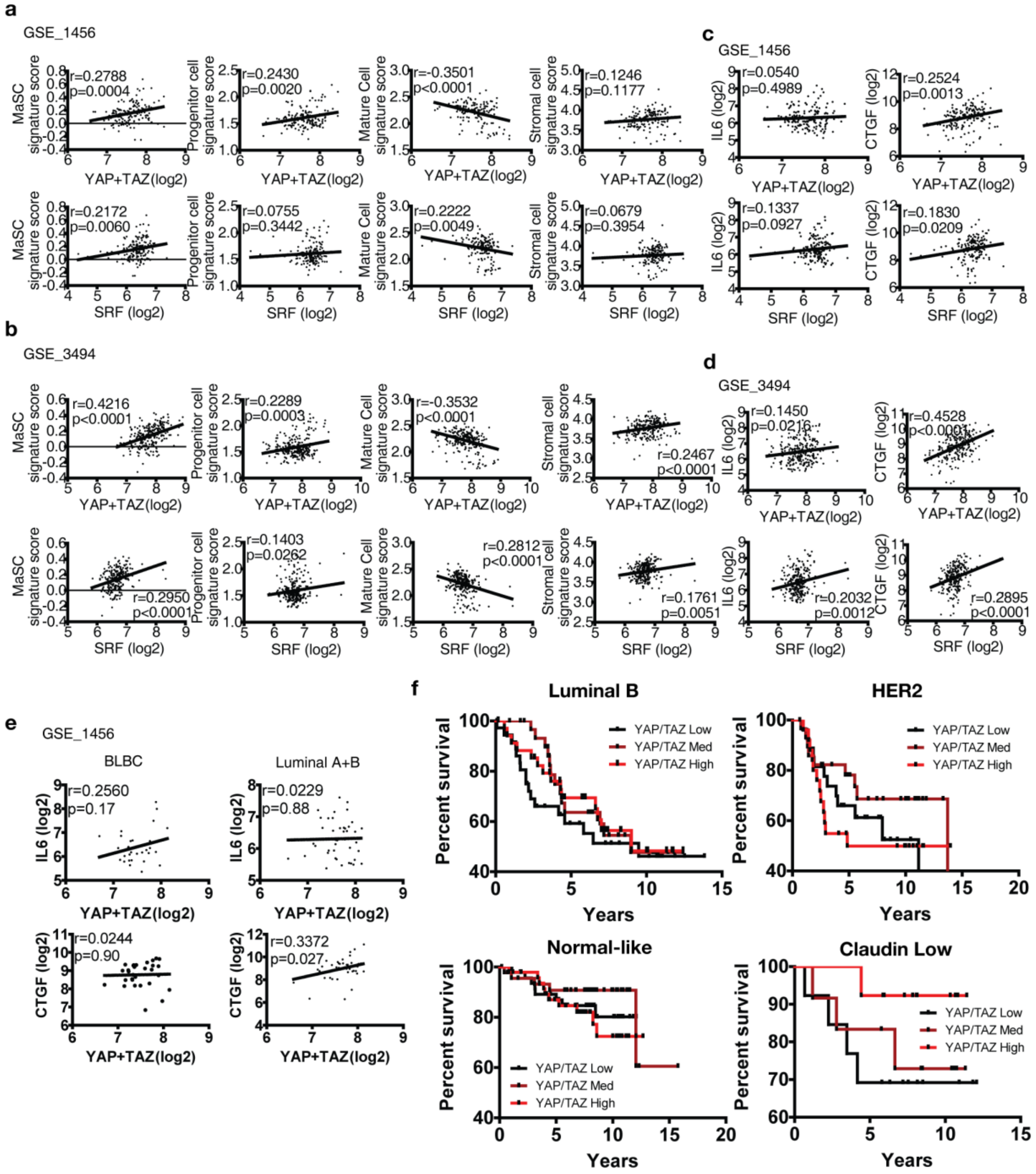

Supplementary Figure 13. YAP/TAZ and SRF are upregulated in basal-type breast cancer and their expression is correlated with a stem/progenitor cell signature and IL6 and CTGF expression levels. (a,b) Analyses of the two independent breast cancer cohorts, GSE1456 (n=159) and GSE3494 (n=251); the approach was similar to that described in Fig. 6e (correlation tested using Pearson's correlation coefficient, r). (c,d) Analysis of the two independent breast cancer cohorts, GSE1456 (n=159) and GSE3494 (n=251); the approach was similar to that used in Fig. 6f (correlation tested using Pearson's correlation coefficient, r). (e) Correlation between YAP/TAZ and IL6 expression in BLBC and luminal-type breast cancer (n>29 for each subtype; correlation tested using Pearson's correlation coefficient, r). (f) Kaplan–Meier curves for other types of breast cancer not listed in Fig. 7g with different levels of YAP/TAZ expression. No subtypes showed a significant correlation (n>38 for each subtype; log-rank test).

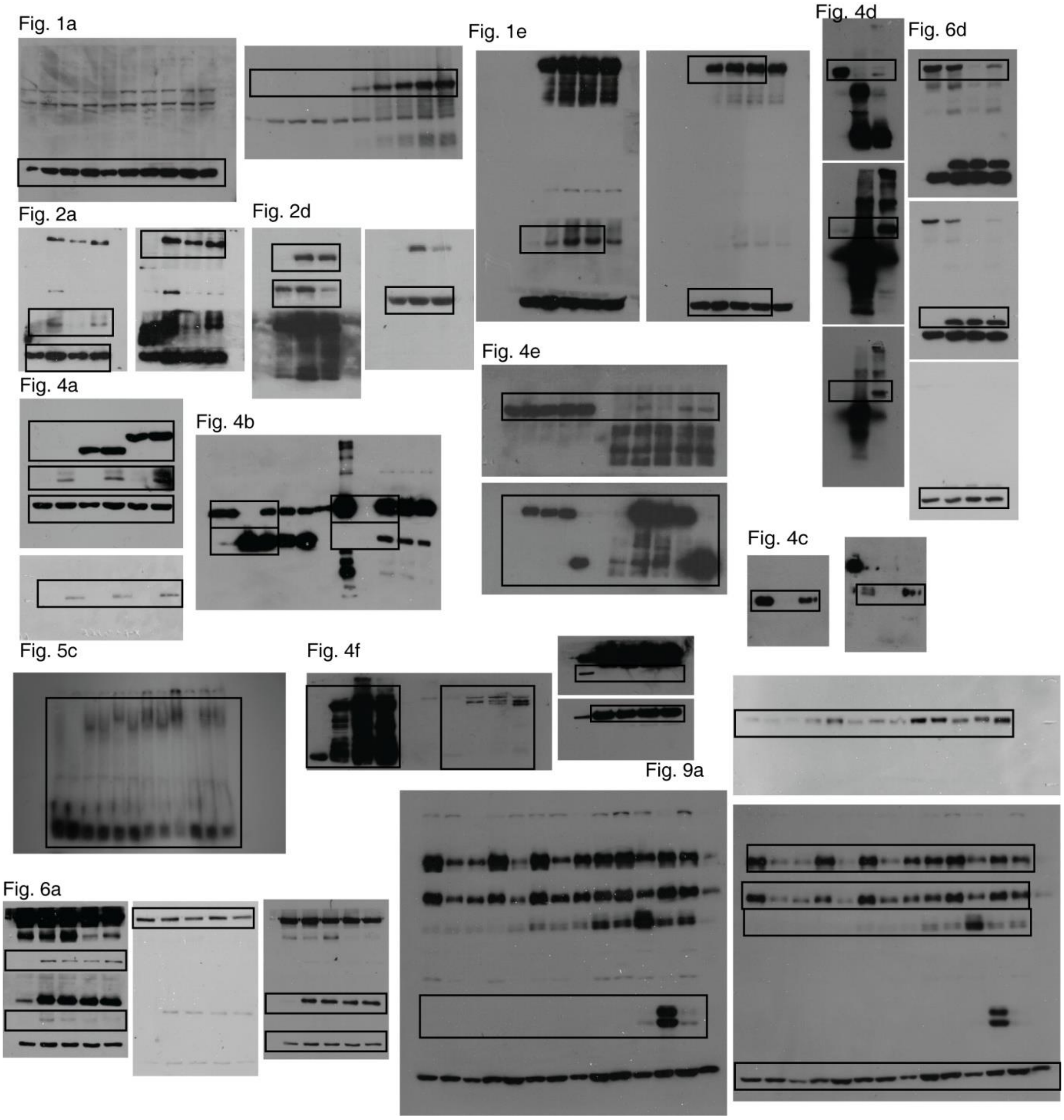

Fig. 1a
Fig. 1e
Fig. 4d
Fig. 6d
Fig. 2a
Fig. 2d
Fig. 4e
Fig. 4a
Fig. 4b
Fig. 4c
Fig. 5c
Fig. 4f
Fig. 9a
Fig. 6a

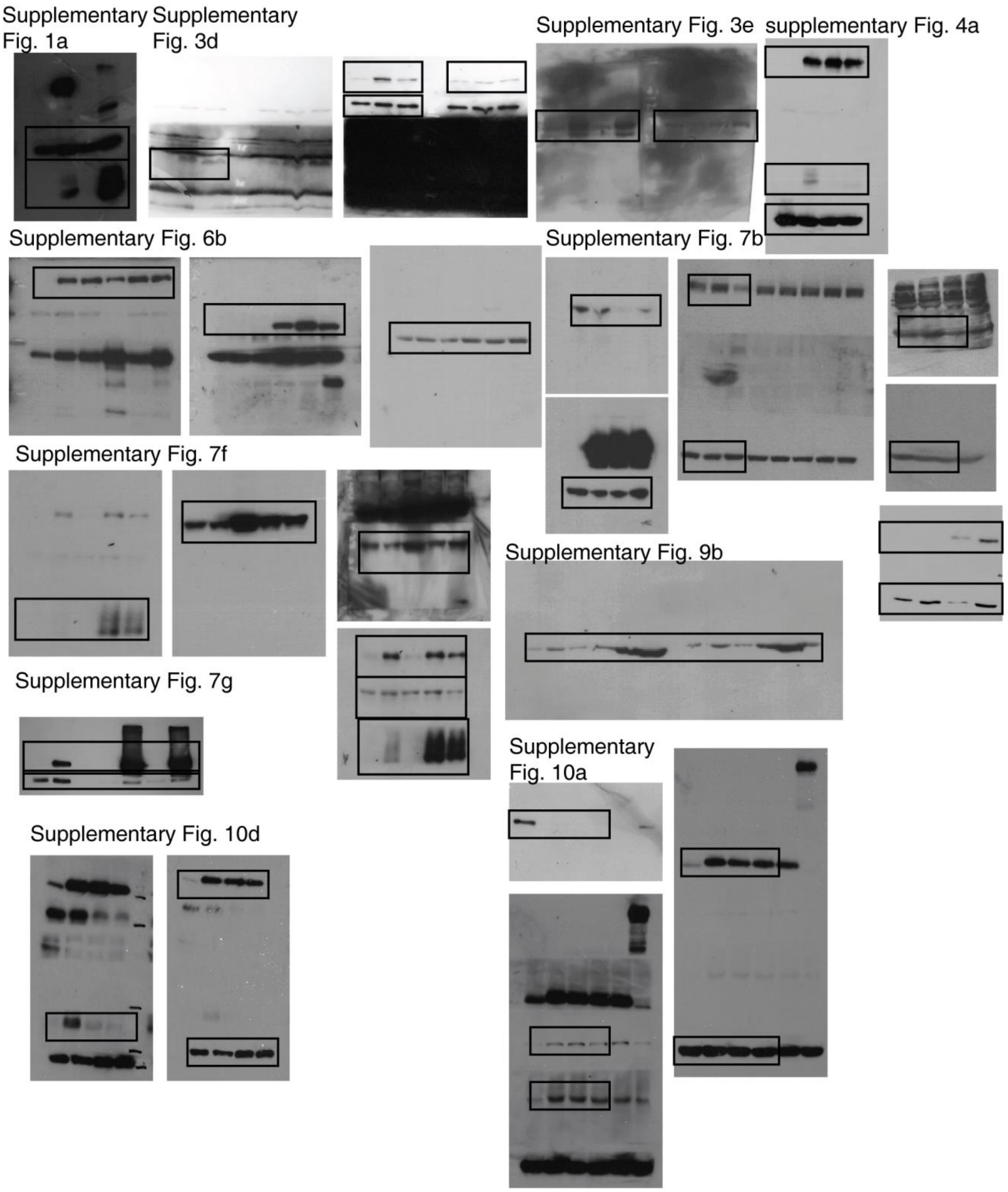

Supplementary Figure 14. Original, uncropped image of blots in main and Supplementary Figures.

Supplementary Table 1. List of primers used for qRT-PCR and ChIP-qPCR

RT-qPCR primers

Human genes

IL6	Forward	AAATTCGGTACATCCTCGACGG
	Reverse	AGGTTCAGGTTGTTTTCTGC
HPRT1	Forward	CCTGGCGTCGTGATTAGTGAT
	Reverse	AGACGTTCAGTCCTGTCCATAA
TEAD1	Forward	ATGGAAAGGATGAGTGACTCTGC
	Reverse	TCCCACATGGTGGATAGATAGC
TEAD2	Forward	GCCTCCGAGAGCTATATGATCG
	Reverse	TCACTCCGTAGAAGCCACCA
TEAD3	Forward	TCATCCTGTCAGACGAGGG
	Reverse	TCTTCCGAGCTAGAACCTGTATG
TEAD4	Forward	GAACGGGGACCCTCCAATG
	Reverse	GCGAGCATACTCTGTCTCAAC
ETS1	Forward	ACCGTGCTGACCTCAATAAGG
	Reverse	CCCCGCTGTCTTGTGGATG
DLL1	Forward	TCCTGATGACCTCGCAACAGA
	Reverse	ACACACGAAGCGGTAGGAGT
ANKRD1	Forward	AGTAGAGGAACTGGTCACTGG
	Reverse	TGGGCTAGAAGTGTCTTCAGAT
CTGF	Forward	ACCGACTGGAAGACACGTTTG
	Reverse	CCAGGTCAGCTTCGCAAGG
THBS1	Forward	CCTGACCGTCCAAGGAAAGC
	Reverse	CCTTTGCGATGCGGAGTCT
SGK1	Forward	AGGATGGGTCTGAACGACTTT
	Reverse	GCCCTTTCCGATCACTTTCAAG

EDN1	Forward	TGTGTCTACTTCTGCCACCT
	Reverse	CCCTGAGTTCTTTTCCTGCTT
ITGB2	Forward	AGTGTGACACCATCAACTGTG
	Reverse	GCACTCGCATACGTTGCAG
CDH1	Forward	ATTTTTCCCTCGACACCCGAT
	Reverse	TCCCAGGCGTAGACCAAGA
CDH2	Forward	TGCGGTACAGTGTAACTGGG
	Reverse	GAAACCGGGCTATCTGCTCG
VIM	Forward	AGTCCACTGAGTACCGGAGAC
	Reverse	CATTTCACGCATCTGGCGTTC
FN1	Forward	AGGAAGCCGAGGTTTTAACTG
	Reverse	AGGACGCTCATAAGTGTCACC
NEDD9	Forward	ATGGCAAGGGCCTTATATGACA
	Reverse	TTCTGCTCTATGACGGTCAGG
SLUG	Forward	TGTGACAAGGAATATGTGAGCC
	Reverse	TGAGCCCTCAGATTTGACCTG
SRF	Forward	CCGGCAAGGCACTGATTCA
	Reverse	CTCATTCTCTGGTCTGTTGTGG
ACTB	Forward	CATGTACGTTGCTATCCAGGC
	Reverse	CTCCTTAATGTCACGCACGAT
MYH9	Forward	CCGCCAAGCCAAGGAAGAA
	Reverse	GGTATTTGTTGTACGGCTCCA

Mouse genes

Il6	Forward	TAGTCCTTCCTACCCCAATTTCC
	Reverse	TTGGTCCTTAGCCACTCCTTC
Gapdh	Forward	AGGTCGGTGTGAACGGATTTG
	Reverse	TGTAGACCATGTAGTTGAGGTCA

ChIP-qPCR primers

Human genomic loci

IL6	Forward	CTGCAAGTTCCCACAGTTCA
	Reverse	CCCACCTTCTTCAAAATCCA
PCDH7	Forward	CGCAACCATCCAAAGTCTG
	Reverse	CCCAGAAAGCCACTCTGTTC
FST	Forward	TAAAGCACTCCGGATCTTGC
	Reverse	TGCGTGCTTTGTAAGTGTCC
PPP2R2B	Forward	ATGTGGAGGCAGAAAACACC
	Reverse	ATGGGAGTAGGCTGCAGAGA
ETS1	Forward	ATTCTCACCTAGACACTGTGC
	Reverse	CCGAACCTCAGTTCCTCCAT
DLL1	Forward	TGCCTAGGAGGGCAATTTAG
	Reverse	CCCCAACCCACAACCTTTAC
CTGF (TEAD binding sequence)	Forward	GGAGTGGTGCGAAGAGGATA
	Reverse	GCCAATGAGCTGAATGGAGT
THBS1	Forward	ACACTCCCACGCAAGAAAAG
	Reverse	GGCCAGGGCATAGGTAGAAG
CYR61 (TEAD binding sequence)	Forward	TTCCAAGCGGTAAAGCATTC
	Reverse	TCCCAAAGTGCTGGGATTAC
CTGF (CArG box)	Forward	TTCCTGCTGTTTGCCTCTTC
	Reverse	TATCCTCTTCGCACCACTCC
CYR61 (CArG box)	Forward	CAAGAATGCTTTGTGGTTGG
	Reverse	GGTGAATCAGACACCAGACG
ANKRD1 (CArG box)	Forward	GGCACTTTTCTATGCAGTTGG
	Reverse	TTCTGGCAGCATATTTCAGC
SRF	Forward	TATGCAAATAAGCGCCCTCT
	Reverse	CCCCCATATAAAGAGATACAATGTT
MYH9	Forward	CTCAAGACGCTCACAATGGA
	Reverse	CTCATGTCATCCCACCACAA
VCL	Forward	CCCGCTGAGGTGATTCTG
	Reverse	GATTCCCGAGCCCTAACG
Gene Desert	Forward	GTTCATCCCAGCACCTGTCT

	Reverse	GTGATGGACCTGGAGCCTAA

Mouse genomic loci

Il6	Forward	ATGAGGGTGTTCCTCCACAC
	Reverse	CCTTGTCAGGCATCAATGG
Dll1	Forward	GGCCCTCCCAATAAACTCAT
	Reverse	CCCCCAACACATAACCTTTA
Ets1	Forward	CTTTCTGGCTGGTAGGCAAG
	Reverse	TGAGAGCTGCCCTTTGTTTC
Thbs1	Forward	CTGGGTGTTTCCAAGGTTTG
	Reverse	CAGGGATCCAGGTAAAAGCA
Gene desert	Forward	CATCAAGGTGCTGGACAAGA
	Reverse	AAAACCTTTGCTCCTGCTGCTGA

Supplementary Reference

1. **Dupont, S. et al. Role of YAP/TAZ in mechanotransduction. *Nature* 474, 179-183 (2011).**
